# The development and maintenance of the mononuclear phagocyte system of the chick is controlled by signals from the macrophage colony-stimulating factor receptor

**DOI:** 10.1186/s12915-015-0121-9

**Published:** 2015-02-19

**Authors:** Valerie Garceau, Adam Balic, Carla Garcia-Morales, Kristin A Sauter, Mike J McGrew, Jacqueline Smith, Lonneke Vervelde, Adrian Sherman, Troy E Fuller, Theodore Oliphant, John A Shelley, Raksha Tiwari, Thomas L Wilson, Cosmin Chintoan-Uta, Dave W Burt, Mark P Stevens, Helen M Sang, David A Hume

**Affiliations:** The Roslin Institute and Royal (Dick) School of Veterinary Studies, The University of Edinburgh, Easter Bush, Midlothian EH25 9RG UK; Zoetis, 7000 Portage Road, Kalamazoo, MI 49001 USA

**Keywords:** Chicken, CSF1, Hematopoiesis, Macrophage, Transgenic

## Abstract

**Background:**

Macrophages have many functions in development and homeostasis as well as innate immunity. Recent studies in mammals suggest that cells arising in the yolk sac give rise to self-renewing macrophage populations that persist in adult tissues. Macrophage proliferation and differentiation is controlled by macrophage colony-stimulating factor (CSF1) and interleukin 34 (IL34), both agonists of the CSF1 receptor (CSF1R). In the current manuscript we describe the origin, function and regulation of macrophages, and the role of CSF1R signaling during embryonic development, using the chick as a model.

**Results:**

Based upon RNA-sequencing comparison to bone marrow-derived macrophages grown in CSF1, we show that embryonic macrophages contribute around 2% of the total embryo RNA in day 7 chick embryos, and have similar gene expression profiles to bone marrow-derived macrophages. To explore the origins of embryonic and adult macrophages, we injected Hamburger-Hamilton stage 16 to 17 chick embryos with either yolk sac-derived blood cells, or bone marrow cells from EGFP^+^ donors. In both cases, the transferred cells gave rise to large numbers of EGFP^+^ tissue macrophages in the embryo. In the case of the yolk sac, these cells were not retained in hatched birds. Conversely, bone marrow EGFP^+^ cells gave rise to tissue macrophages in all organs of adult birds, and regenerated CSF1-responsive marrow macrophage progenitors. Surprisingly, they did not contribute to any other hematopoietic lineage. To explore the role of CSF1 further, we injected embryonic or hatchling CSF1R-reporter transgenic birds with a novel chicken CSF1-Fc conjugate. In both cases, the treatment produced a large increase in macrophage numbers in all tissues examined. There were no apparent adverse effects of chicken CSF1-Fc on embryonic or post-hatch development, but there was an unexpected increase in bone density in the treated hatchlings.

**Conclusions:**

The data indicate that the yolk sac is not the major source of macrophages in adult birds, and that there is a macrophage-restricted, self-renewing progenitor cell in bone marrow. CSF1R is demonstrated to be limiting for macrophage development during development *in ovo* and post-hatch. The chicken provides a novel and tractable model to study the development of the mononuclear phagocyte system and CSF1R signaling.

**Electronic supplementary material:**

The online version of this article (doi:10.1186/s12915-015-0121-9) contains supplementary material, which is available to authorized users.

## Background

The mononuclear phagocyte system is a family of cells consisting of committed progenitors, circulating monocytes and tissue macrophages [1,2]. The prevailing view that blood monocytes give rise to tissue macrophages in the steady state has been challenged by recent evidence in the mouse that phagocytic cells arising in the yolk sac can populate tissues during development and can be maintained through local proliferation [2,3]. The generation of phagocytes in the yolk sac and their infiltration of the embryo has been demonstrated in the chick [4,5] and in the mouse ([1-3,6-8] and references therein). Yolk sac-derived macrophages in the chick were shown to enter the developing central nervous system independently of vascularization [4]. Their origin was confirmed through the use of chick-quail yolk sac chimeras [5]. However, the inter-specific chimera system used did not permit full development, so there was no evidence from the chick system as to whether the yolk sac-derived cells were retained post-hatch.

The hematopoietic growth factors macrophage colony-stimulating factor (CSF1) and interleukin 34 (IL34) control the proliferation, differentiation and development of mammalian mononuclear phagocytes [9,10], acting through a common receptor, CSF1R. All three molecules are conserved in birds [11]. A critical control element in the first intron of the *Csf1r* locus, termed the fms intronic regulatory element (FIRE) in mammals [12] is also conserved in birds [11]. *Csf1r* mRNA is detected in the earliest phagocytes in the mouse yolk sac [12,13]. The extreme macrophage deficiency and developmental abnormalities seen in a *Csf1r*^-/-^ mouse embryo [14,15] suggest that CSF1 and IL34 control embryonic macrophage differentiation and, in turn, that these cells are required for normal development.

The contribution of cells that originate in the yolk sac to tissue macrophage populations has been assessed in mice through the use of inducible lineage tracers, and the impact of the knockout of the transcription factor, *c-myb* [2,3,13,16,17]. However, such studies depend upon the assumption that knockouts and inducers such as tamoxifen do not themselves alter the contribution of the yolk sac by compromising definitive hematopoiesis [3]. The chick has been used extensively in developmental biology because of the ease with which cells and tissues can be physically transplanted to allow fate-mapping, an approach made even more straightforward by our development of ubiquitous enhanced green fluorescent protein (EGFP)-expressing chicken lines [18,19]. In the current study we examine the origins of tissue macrophages during embryonic development and the importance of CSF1 in the control of macrophage proliferation and differentiation in the chicken. The results confirm that CSF1 is a regulator of the chick mononuclear phagocyte system *in vivo*. They also suggest that yolk sac-derived macrophages in the chick are relatively short-lived and are substituted by bone marrow-derived macrophages (BMDM) during development.

## Results

### Comparative RNA-sequencing analysis of CSF1-stimulated bone marrow-derived macrophages and embryonic RNA

Macrophages are a major cell population in the developing mouse embryo, as evident from both *in situ* hybridization of *Csf1r* and other macrophage-related mRNAs [6], and the location of a macrophage-specific *Csf1r-*EGFP reporter transgene [12]. The visualization of cells expressing *CSF1R* reporter genes ([20,21] and see below) in the chick suggests that embryonic macrophages are just as abundant in this species, but there is limited information on macrophage-restricted mRNAs to enable their characterization.

**Figure 1:**
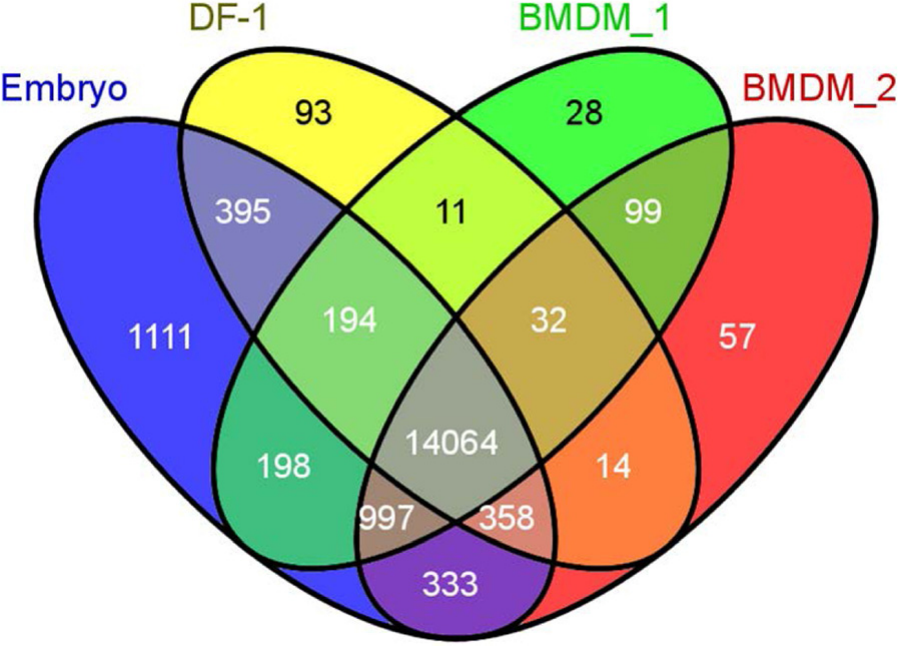
**Comparison of gene expression of chicken bone marrow-derived macrophages, DF1 fibroblasts and day 7 embryo based upon RNA sequencing.** RNAseq of RNA from the four sources (including two independent biological replicates of BMDM) was carried out and results analyzed as described in Methods. This Venn diagram shows the relationship between the datasets. The numbers represent the numbers of unique gene annotations detected in each RNAseq library based upon a 1 tag per million threshold. BMDM, bone marrow-derived macrophages.

To reveal macrophage-enriched transcripts, we used RNA-sequencing (RNAseq) to compare the mRNA profiles of BMDM grown in CSF1 with a pool of E7 embryos. The fibroblast line DF1 was used as a negative control. We identified transcripts with unique annotations, and with an expression threshold of 1 tag/million (tpm) in the embryo, and created a Venn diagram of overlapping expression (Figure 1). Around 75% of transcripts detected at 1 tpm were represented in all of the libraries. At this threshold, 30% to 40% of transcripts were detected in only one of the two BMDM libraries. We focused on two sets: 997 transcripts that were detected in both BMDM libraries and embryo but not in DF-1, and 99 that were detected only in the two BMDM libraries (Additional file 1: Tables S1, S2 and S3). The tables show the relative expression in DF1 cells and embryo compared with BMDM. *CSF1R* mRNA was detected in embryos at around 2% of the level found in pure BMDM. Assuming similar levels of expression of *CSF1R* mRNA in tissue macrophages and BMDM, this would suggest that macrophages contribute around 2% of the total mRNA, which is consistent with their apparent abundance. The macrophage-specific transcription factor PU.1 (*SFPI1*) had a similar relative enrichment to *CSF1R*, whilst several other myeloid transcription factors (*MAFB*, *CEBPE*, *TFEC*, *RUNX1*) were less enriched in the BMDM relative to the embryo, most probably because they are expressed in other hemopoietic cells (Additional file 1: Table S1). The macrophage-enriched list also includes many known macrophage-expressed surface proteins, including *CSF2RB*, *CCR2*, *TREM2*, *CD36*, *MRC1*, *P2RY2*, *STAB1* and *TLR7*, detected by network analysis of large mouse, pig and human datasets [22-24]. The 99 macrophage-expressed genes that were below the 1 tpm in embryo are, in fact, detectable. As shown in Additional file 1: Table S3, they were all highly enriched in BMDM relative to DF1 cells, and include many genes that are macrophage-enriched in mice (for example, *LY86*, *CTSS*, *C1Q*, *CSF3R*).

### The origins of embryonic macrophages

**Figure 2:**
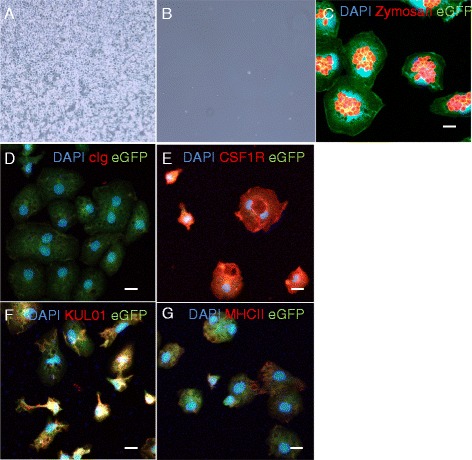
**Circulating yolk sac-derived cells from HH16 to HH17 stage embryos can be differentiated into macrophages** ***in vitro*** **. (A, B)** Twenty microliters of whole blood from five HH16 to HH17 stage embryos was cultured with **(A)** or without **(B)** 350 ng/ml chicken CSF1-Fc for 14 days. Note the confluent monolayer of cells in panel **A**, and the absence of any surviving cells in panel **B**. 20× magnification. **(C)** Shows the ability of cells derived by cultivation from EGFP^+^ yolk sac donors to internalize fluorescent zymosan particles. **(D, G)** Shows the same cell populations immunostained with control **(D)**, anti-CSF1R **(E)** anti-KUL01 **(F)** or anti-MHCII **(G)**. Scale bars = 10 μm.

Embryonic macrophages are first observed in the yolk sac at Hamburger-Hamilton stage (HH) 13, prior to the first appearance of progenitors of chicken definitive hematopoietic stem cells in the ventral floor of the dorsal aorta around HH16 [25,26]. Recognizable blood islands containing Runx1^+^ hematopoietic progenitors have been detected in HH5 embryos but the cells expressing a *CSF1R*-reporter gene appeared in the yolk sac at HH13. These cells were confined to the lumen of primitive blood vessels and their numbers rapidly escalated in the circulation [21]. The pattern of emergence is consistent with previous reports of the earliest appearance of macrophages in the chicken embryo [5]. We tested the potential of circulating yolk sac-derived cells to differentiate into macrophages *in vitro* by culturing whole blood from HH16 to HH17 embryos. In the absence of growth factors, these circulating yolk sac-derived cells failed to survive in culture, whereas they rapidly differentiated into adherent cell monolayers in the presence of CSF1 (Figure 2A,B). The resulting cells were identified as macrophages based on their phagocytic capacity and expression of CSF1R, MHCII and KUL01 (a chicken macrophage marker; Figure 2C-G). Circulating yolk sac-derived cells were found to have a high proliferative capacity, with macrophages derived from 20 μl of whole blood expanding to approximately 90% confluence in a T80 flask after 21 days in culture.

**Figure 3:**
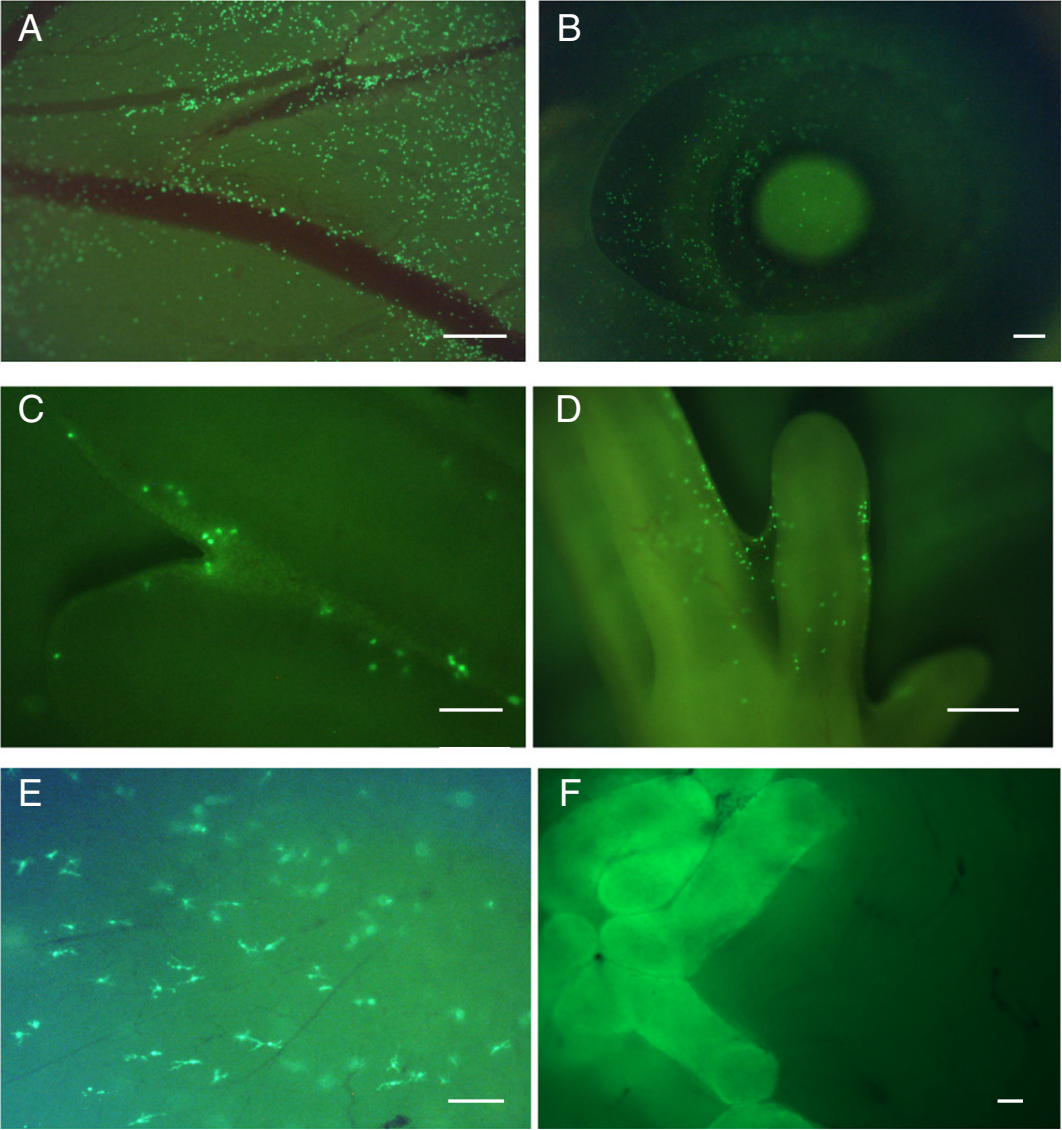
**Embryonic yolk sac-derived cells give rise to macrophages in the embryo.** Cells were isolated from the blood of EGFP^+^ embryos at 3 days of development (HH16 to HH17), prior to the onset of definitive hematopoiesis (which occurs around HH17 to HH19 [26]), and injected into the yolk sac circulation of age-matched non-transgenic recipients. The images are representative whole mounts at different times and locations during subsequent development of separate individual birds. **(A)** Day 6 yolk sac blood vessels; note the extensive infiltration of the yolk sac by the transferred cells. **(B)** Day 10 eye. **(C)** Day 7 inter-digit region. **(D)** Day 8 foot. **(E)** EGFP^+^ microglial cells in the brain of a chick on the day of hatch. **(F)** Ubiquitous expression of EGFP in seminiferous tubules of a 16-week-old male recipient demonstrates successful establishment of germ cell chimerism from donor primordial germ cells. In the same animal, there were no detectable EGFP cells in any other location viewed as a whole mount. Scale bars A = 200 μm, B = 500 μm, C = 200 μm, D = 500 μm, E = 100 μm, F = 100 μm.

**Figure 4:**
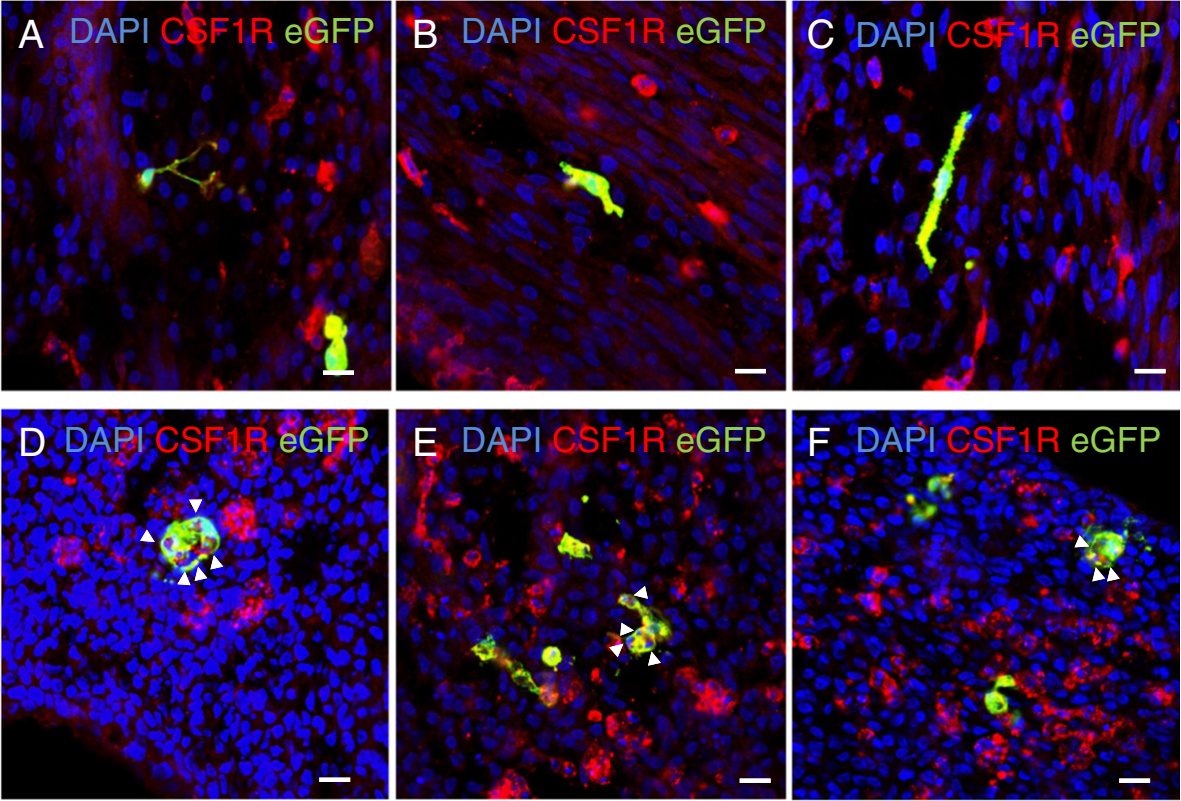
**Cells derived from transferred yolk sac progenitors express surface CSF1R.** Cells were isolated from the blood of EGFP^+^ embryos at 3 days of development (HH16), prior to the onset of definitive hematopoiesis (which occurs around HH17 to HH19 [26]), and injected into the yolk sac circulation of age-matched non-transgenic recipients. Embryos were incubated until stage HH33 (day 8 of development). The identity of the EGFP^+^ cells derived from the donor transfer was confirmed by immunolocation of specific antigens. **(A-C)** Show different sections of embryonic mesoderm from three separate recipients immunostained for CSF1R. Note that the all EGFP^+^ cells are also positive for surface CSF1R (yellow) and exhibit a diverse range of morphologies. **(D-F)** Show sections of the limb bud inter-digit region immunostained for CSF1R. Note again that all EGFP^+^ cells are also CSF1R^+^. In each case, the labeled cells contain numerous DAPI-stained fragments (arrow heads) indicative of the uptake of apoptotic cells. Scale bars = 10 μm.

To test the potential for circulating yolk sac-derived cells to differentiate into macrophages *in vivo* we injected blood from ubiquitously EGFP-expressing stage HH16 to HH17 embryos into the dorsal aorta of age-matched wild-type embryos. Donor-derived EGFP^+^ cells were observed in the yolk sac in all embryonic stages tested and in newly hatched chicks. Figure 3 shows representative images of a number of locations in embryos receiving transferred cells at different times of subsequent development. EGFP^+^ cells apparently (re)entered the yolk sac compartment (Figure 3A). Figure 3B shows the large number of EGFP^+^ cells infiltrating the eye, a site of major embryonic macrophage infiltration detected with the *CSF1R*-EGFP transgene previously [21], and also observed in mouse *Csf1r*-EGFP transgenics [12]. As expected, and observed previously in the mouse [6,12], EGFP^+^ cells were particularly concentrated in regions of programmed cell death, such as the inter-digit region of the limb buds (Figure 3C,D). Finally, consistent with the reported ability of yolk sac-derived cells to give rise to microglia [13,16,17], cells with the morphology of microglia were still evident in the brain of all birds examined at the time of hatch (Figure 3E). We confirmed the phenotype of EGFP^+^ cells in recipient embryos by confocal microscopy. EGFP^+^ cells were variable in morphology, ranging from highly ramified cells in the embryonic mesenchyme to rounded cells in the inter-digit region of limb buds (Figure 4). All EGFP^+^ cells were also labeled by immunohistochemistry with anti-CSF1R, and rounded EGFP^+^ CSF1R^+^ cells in the inter-digit region contained the condensed and fragmented nuclei of apoptotic cells. Hence, the progeny of the transplanted donor cells were functionally phagocytic *in vivo* (Figure 4D-F).

To test the potential for circulating yolk sac-derived cells to contribute to the adult macrophage pool, we again injected blood from ubiquitously EGFP-expressing stage HH16 to HH17 embryos into the dorsal aorta of age-matched wild-type embryos. In three separate experiments a total of 54 recipient embryos were injected; 29 chickens survived until sexual maturity (54% survival rate at 16 weeks of age). In newly hatched chicks, occasional highly ramified EGFP^+^ cells derived from the yolk sac donors were still detectable in the brain. By contrast, no evidence of EGFP fluorescence, compared with non-transgenic birds, was detected in any tissue of any adult bird including the brain when viewed as whole mounts or in sections. As a positive control for effective transfer in these hatched birds, EGFP^+^ cells were detected in the testes of recipient males (Figure 3F), indicating the presence of cells derived from donor primordial germ cells that were co-transferred along with the yolk sac macrophages in blood derived from stage HH16 to HH17 embryos [27].

To test the differentiation potential of definitive hematopoietic cells under the same protocol, we injected unfractionated bone marrow from newly hatched EGFP^-^expressing chicks into the circulation of stage HH16 to HH17 non-transgenic chicken embryos, before the appearance of the intra-aortic hematopoietic stem cell clusters. The workflow for this experiment is shown in Additional file 2: Figure S1. In each of three experiments, 25 embryos were injected, and hatch rates varied between 36% and 60%. As with the embryonic cell adoptive transfers, the injected embryos were examined for the presence of EGFP-expressing cells of donor origin at various stages of embryonic development, and some were also taken to hatch. Tissues from these bone marrow chimeras were collected on hatch day, five 5 of age and 6 weeks of age for similar examination.

**Figure 5:**
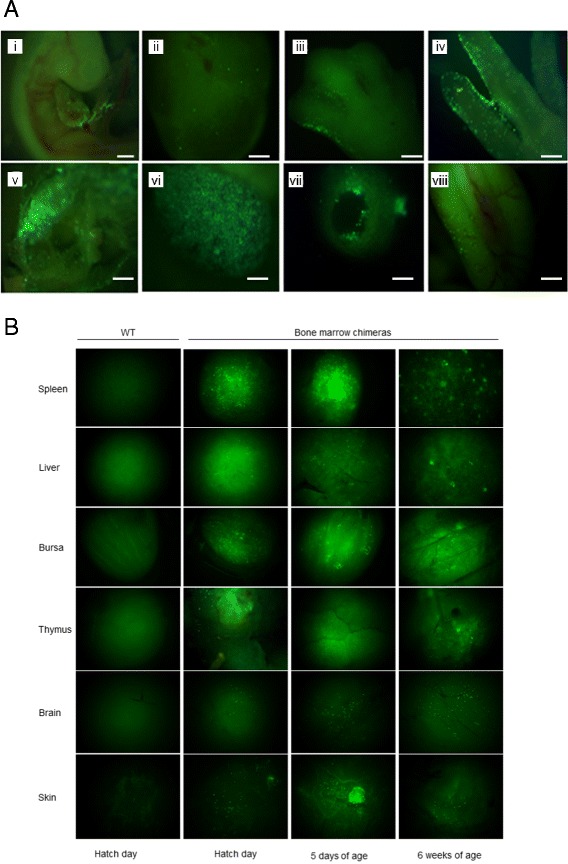
**Bone marrow progenitors give rise to macrophages in the embryo and in adult birds.** Bone marrow cells from newly hatched EGFP^+^ donors were injected into the yolk sac circulation of HH16 non-transgenic recipients as described in Methods. **(A)** Images are representative whole mounts taken under UV illumination at different times and locations during subsequent development, and in different recipients, to provide an indication of the ability of the transplanted cells to populate all locations in the embryo: (i) whole embryo-E4; (ii) brain cross-section-E5; (iii) hindlimb-E7; (iv) hindlimb digits-E10; (v) chorioallantoic membrane-E12; (vi) spleen-E13; (vii) femur cross section-E13; (viii) intestine-E14. **(B)** Images are representative whole-mount images of the spleen, liver, bursa of Fabricius, thymus, brain and skin of representative wild-type and EGFP-bone marrow chimeras at day 0, day 5 and 6 weeks of age as indicated. Scale bars = 200 μm.

Donor bone marrow-derived EGFP^+^ cells invaded the whole embryo as shown in Figure 5A. The distribution of EGFP-expressing cells in the host embryo was comparable to that observed in *CSF1R*-EGFP transgenic chick embryos [21], including in the forming digits (Figure 5A; panels iii and iv). The aggregation of macrophages to form primordia, detected with the *CSF1R*-EGFP or *CSF1R*-mApple reporters, is the first sign of the future location of organized lymphoid organs in the chick [21]. The bone marrow donor-derived EGFP-expressing cells also aggregated in lymphoid tissue primordia of the recipient embryo (for example, the spleen in Figure 5A, panel vi). The distribution of donor EGFP-expressing cells in tissues was examined in post-hatch chimeras by whole mount fluorescence imaging (Figure 5B). In contrast with the loss of labeled cells in chicks injected with yolk sac-derived cells, EGFP^+^ cells were so abundant throughout all the tissues examined (that is, brain, skin, liver, spleen, thymus, bursa, bone marrow and blood) that they were easily detected in whole mounts. Furthermore, these cells from the donor were still present in the tissues at 6 weeks of age, which was the latest time point examined. They were particularly concentrated in the spleen and bursa of Fabricius but less so in the liver, which is not a major site of hematopoiesis during avian development [28]. The donor-derived cells were also detected in the brain and skin of all the chimeras generated. The presence of EGFP-expressing cells in the thymus of the chimeras implies that bone marrow donor-derived progenitors were still circulating at 6.5 days of incubation, which is the time of the first influx of hematopoietic cells into the chicken thymus. The thymus ontogeny involves a cyclic succession of receptive and non-receptive periods for stem cell entry into the embryonic and early postnatal thymus [29]. To confirm the identity of the bone marrow-derived donor cells that persisted in the brains of the chimeras, coronal sections of the right telencephalon were stained for expression of the leukocyte marker CD45 and for CSF1R. All of the EGFP^+^ cells also expressed CD45 and CSF1R, and had the characteristic ramified morphology of microglial cells. Additional file 2: Figure S2 shows one example.

**Figure 6:**
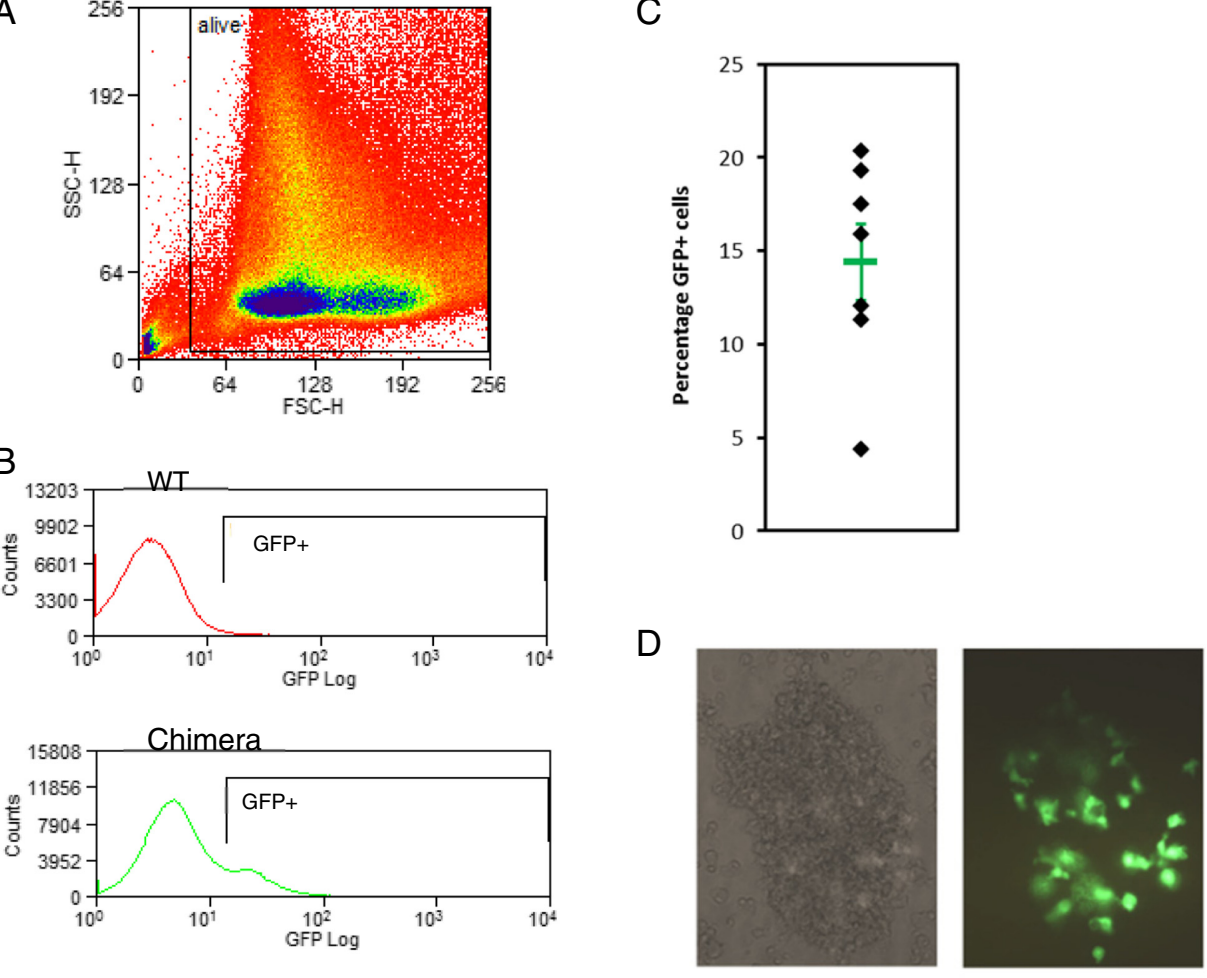
**Flow cytometry analysis of bone marrow chimeras.** Bone marrow cells from EGFP^+^ donors were injected into the yolk sac circulation of HH16 non-transgenic recipients as described in Methods. Dissociated bone marrow from 6-week-old chimeras was analyzed by fluorescence-activated cell sorting. **(A)** A representative scatter plot of the cells; more complex than mammalian marrow because both red cells and thrombocytes are nucleated. **(B)** A representative EGFP profile of control and EGFP-chimeric marrow. **(C)** Percentage chimerism across multiple birds analyzed; the average is 13.72 ± 2.02% (corrected for the small number of autofluorescent cells in control marrow). **(D)** Bone marrow cells from the newly hatched chimeric birds were cultivated in chicken CSF1 for 7 days to produce BMDM. In these cultures, large clusters of EGFP^+^ cells, approximately consistent with the level of chimerism, were detected. Images are a representative cluster in which all of the cells are EGFP^+^ (the apparent intensity varies within the cells in the cluster, depending upon how spread they are on the substratum).

The EGFP^+^ macrophages in post-hatch chimeric birds might derive from local self-renewal of macrophages seeded during development, or from population of the hematopoietic progenitor pools, or both [2,3]. We therefore examined whether injected EGFP+ bone marrow cells isolated from newly hatched chicks could contribute to the progenitor pool in the marrow of recipients. The bone marrow of 6-week-old chimeras contained 10% to 20% of EGFP^+^ cells (Figure 6). The EGFP^+^ cells were predominantly large and granular, and expressed high levels of CD45, consistent with their identity as macrophages (not shown). This restricted profile suggests that the donor cells can only give rise to the macrophage lineage in the recipient marrow. To confirm the contribution of the donor EGFP^+^ cells to macrophage progenitors, chimeric marrow was cultured in presence of chicken CSF1 for 7 days. A substantial proportion of the resulting BMDM expressed EGFP. The positive cells occurred in large clusters in the cultures, suggestive of colonies derived from a progenitor (Figure 6).

The level of chimerism in spleens of recipient birds at 6 weeks of age was more difficult to assess. There was an apparent increase in background fluorescence in the chimeric birds. On flow cytometry, the entire fluorescence curve of large granular cells within the spleen was shifted somewhat to the right, perhaps due to the uptake of EGFP from dying cells (Additional file 2: Figure S3A). Frozen sections of paraformaldehyde (PFA)-fixed chimeric spleens were stained using an antibody against the cell surface antigen Bu-1, which is expressed on B cells and a subset of macrophages [30]. None of the EGFP^+^ cells were positive for Bu-1. The level of chimerism in the large granular cell fraction was as high as 45% in two of the birds examined in detail. Sustained chimerism was confirmed in the bursa, where the background EGFP shift was not observed (Additional file 2: Figure S3B). In this organ, around 13% of the large granular cells were unequivocally EGFP^+^, whereas amongst small lymphocyte-like fractions the proportion was only 1% to 2%. As in the spleen, the EGFP^+^ cells in tissue sections did not stain for Bu-1; indeed, the positive cells were excluded from the B cell follicles. Conversely, all of the EGFP^+^ cells in both spleen and bursa were stained with our recently described antibody against CSF1R, and were not morphologically distinct from the large pool of stellate interstitial CSF1R^+^ macrophages [20].

### The responsiveness of embryonic macrophages to CSF1

The ability of donor bone marrow cells to proliferate and differentiate when transplanted into the chick embryo indicates that there is a macrophage-trophic environment. Consistent with this view, *in situ* hybridization and quantitative RT-PCR demonstrated that both *CSF1* and *IL34* mRNA were highly expressed in the embryo, especially in the head (and, in the case of *IL34*, the neural tube) at the time of proliferation of the macrophage populations expressing *CSF1R* mRNA (Additional file 2: Figure S4A,B). To determine whether the availability of CSF1R ligands is limiting for macrophage development, we examined the effect of supplementing their availability by direct microinjection. For this purpose, we developed a chimeric chicken CSF1-Fc fusion protein (see Methods). A similar construct for pig CSF1 was found to increase the circulating half-life, because it increases the size of the protein above the renal clearance threshold [31]. We assayed the chicken CSF1-Fc using a chicken CSF1R-expressing Ba/F3 line described previously [11] and found that the addition of the Fc component did not alter the relative activity (Additional file 2: Figure S5). The MacGreen (*CSF1R*-EGFP) transgenic reporter chick embryos [21] provided a convenient assay of the effect of CSF1. The chicken CSF1-Fc conjugate was injected into the neural tube of MacGreen embryos at HH21 and the number of *CSF1R* transgene-expressing cells was evaluated 36 hours later. Definitive hematopoietic stem cells appear in the aorta-gonad-mesonephros region at HH19 so the exogenous CSF1 injected at this stage might act on both yolk sac and definitive monocyte macrophages. There was no consistent difference in the gross appearance or size of whole embryos injected with chicken CSF1-Fc and PBS within the time frame examined. However, when whole embryos were viewed as whole mounts, there was a very obvious increase in EGFP fluorescence to the extent that the injected embryos were bright green (Additional file 2: Figure S6A). The apparent increase in EGFP fluorescence was not focused at the site of injection, but was manifest throughout the chicken CSF1-injected embryos. Comparison of sagittal sections through the body and of the footpad (Additional file 2: Figure S6B) confirmed that the injected chicken CSF1-Fc did not change the localization or distribution of EGFP^+^ cells, and that the increased EGFP fluorescence was associated with an apparent increase in the numbers of EGFP^+^ macrophages in the same locations.

### Control of macrophage production in newly hatched birds by CSF1

Increased CSF1 can cause a large expansion of the monocyte and tissue macrophage populations in mammals, but this has never been studied in birds. We therefore wished to determine whether the availability of CSF1 remained limiting for the proliferation and differentiation of macrophages in post-hatch life in the chicken. For this purpose, the chicken CSF1-Fc was injected into MacRed transgenic reporter chicken hatchlings, in which *CSF1R* control elements drive expression of mApple. There are limited alternative markers for embryonic chick macrophages. Expression of the *CSF1R* reporter genes in chick was shown to be coincident with CD45, surface CSF1R and staining with lysotracker. Their location in regions of high cell death and stellate morphology resemble the distribution of macrophages seen in mouse embryos. In adult birds, all KUL01^+^ cells, including blood monocytes and tissue macrophages also express the reporter gene, but KUL01 is not a useful marker in the embryo [21]. MacRed hatchlings were dosed subcutaneously with 50 μg chicken CSF1-Fc or PBS on hatch day (day 0), day 1, day 2 and day 3, then sacrificed 24 hours after the final injection.

**Figure 7:**
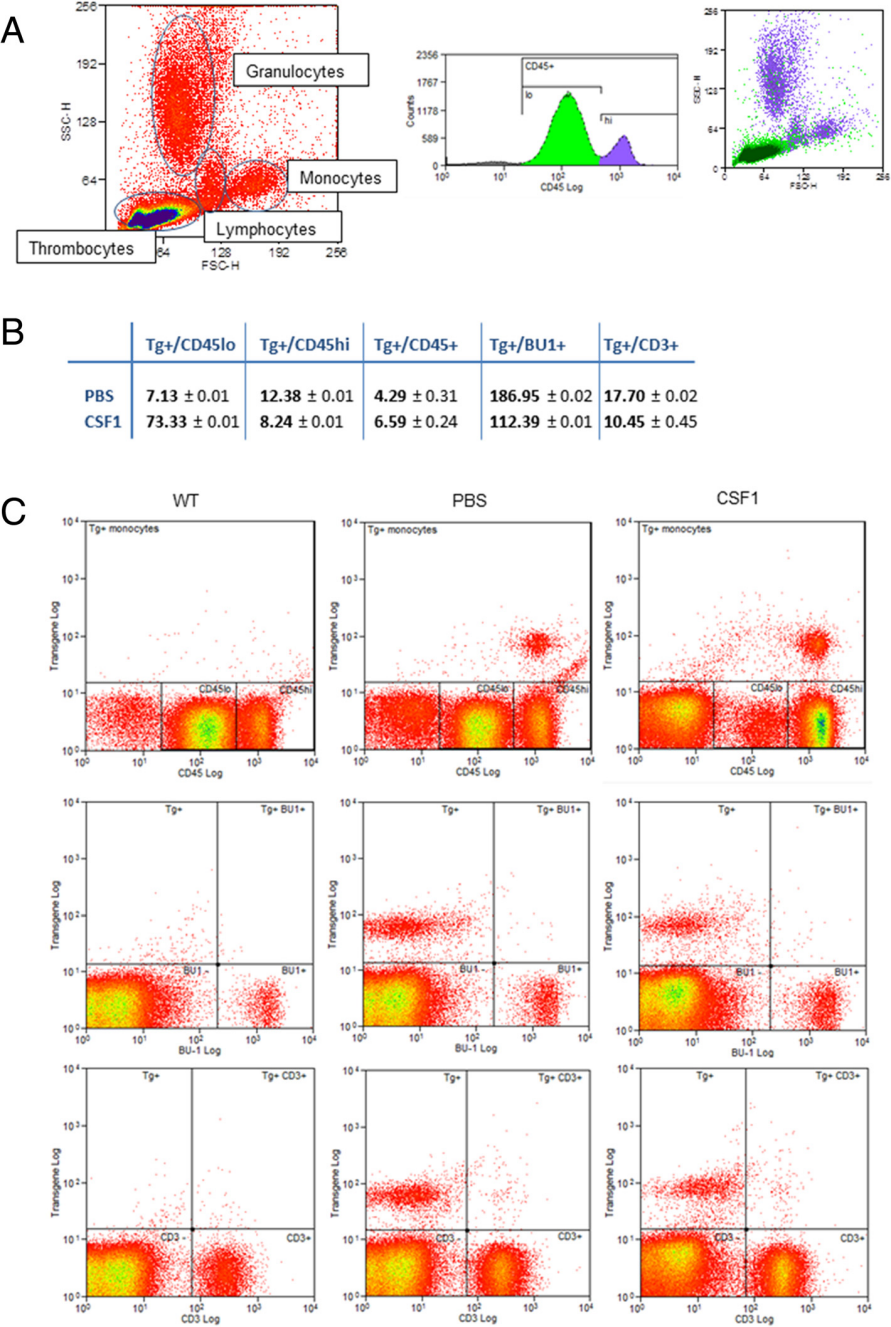
**The effect of chicken CSF1-Fc on blood cell profiles in newly hatched birds.** MacRed (*CSF1R*-mApple) birds were dosed subcutaneously with 50 μg chicken CSF1-Fc or PBS on hatch day (day 0), day 1, day 2 and day 3, then sacrificed 24 hours after the final injection. Blood was collected into heparinized tubes, and separated by density gradient centrifugation as described in Methods. The cells were stained with anti-CD45, anti-CD3 or anti-BU-1, and analyzed by flow cytometry. **(A)** Representative FACS profiles of marker expression used to calculate the values in **(B)**. **(B)** Mean monocyte number (transgene-expressing cells) compared to mean thrombocyte number (CD45^lo^), mean leukocyte number (CD45^hi^), mean thrombocyte + leukocyte number (CD45^+^), mean B cell number (BU-1^+^) or mean T cell number (CD3^+^) for each treatment group. **(C)** Panels show representative FACS plots for the peripheral leukocyte populations from non-transgenic wild-type birds, or PBS or chicken CSF1-Fc-treated MacRed birds as indicated, stained for CD45 (upper panels), Bu-1 (middle panels) or CD3 (lower panels). The transgene (mApple) is on the y-axis, detecting the transgenic macrophages. PBS, treated with phosphate-buffered saline; WT, wild-type.

CSF1 treatment caused a leukocytosis, with a two- to three-fold increase in total white cell count in all treated birds (not shown). The fluorescence-activated cell sorting (FACS) profiles of the leukocytes are shown in Figure 7A. The *CSF1R*-mApple transgene provides a convenient marker for the monocytes. At first glance, it appears that the proportion of monocytes in the blood increased in response to chicken CSF1-Fc, but this is actually entirely due to the proportional loss of thrombocytes. In the chicken, these cells are nucleated but express relatively low levels of CD45 compared to other leukocytes. As shown in Figure 7B, the ratio of transgene-positive monocytes to CD45^lo^ thrombocytes was greatly increased by the chicken CSF1-Fc treatment. Conversely, the leucocytosis was not selective for monocytes. Indeed, the ratio of monocytes to B cells, T cells, or total CD45^hi^ cells was significantly reduced in the treated birds. A similar pattern has been seen in mammals, including primates, where CSF1 treatment caused thrombocytopenia, but increased all the white cell populations [32].

**Figure 8:**
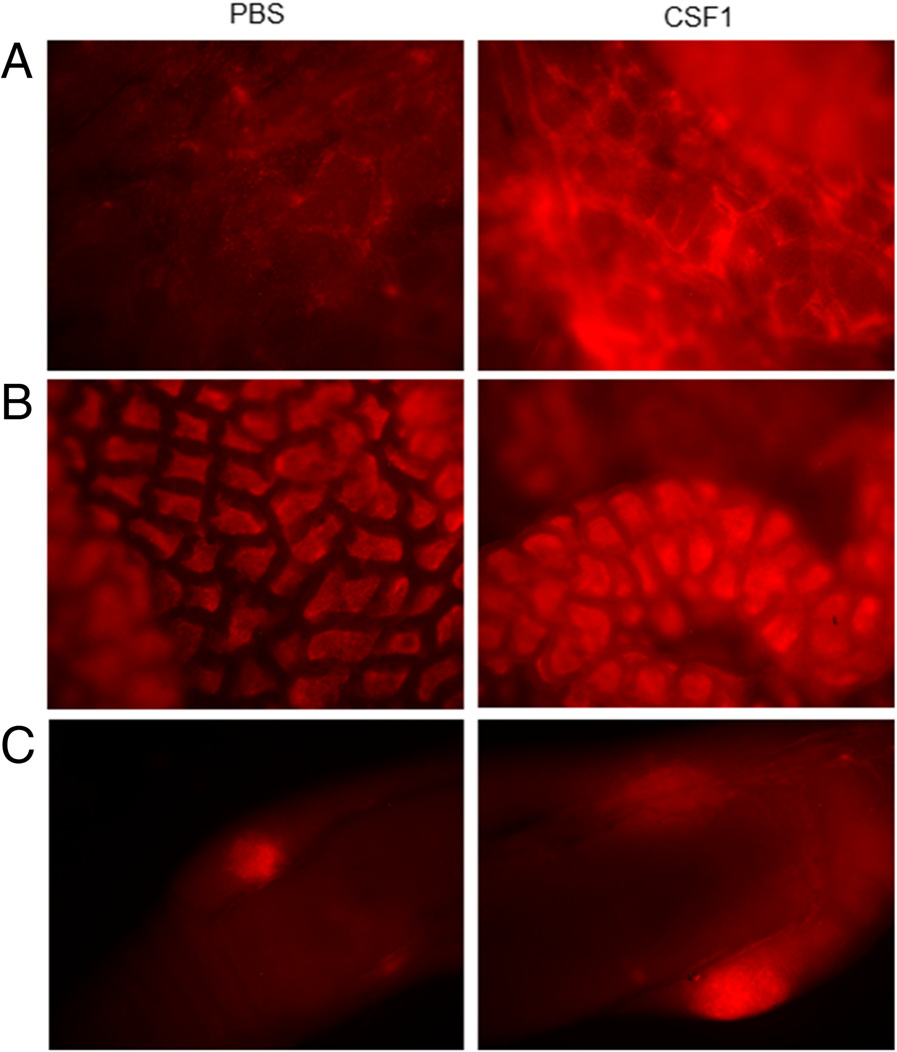
**The effect of chicken CSF1-Fc in macrophage concentration in tissues detected by whole mount imaging of MacRed chick.** Hatchlings were dosed subcutaneously with 50 μg chicken CSF1-Fc or PBS on hatch day (day 0), day 1, day 2 and day 3, then sacrificed 24 hours after the final injection. Tissues were collected quickly following the sacrifice of the chicks, and put in ice-cold PBS for imaging under UV light using a red fluorescence filter for the same exposure time. **(A)** Skin biopsy, 40× magnification. **(B)** Inner surface of the rectum, split open, 80× magnification. **(C)** Cecum, 12.5× magnification. PBS, phosphate-buffered saline.

As in the chicken CSF1-Fc-treated embryos described above, whole mounts of tissues of the chicken CSF1-Fc-treated birds could be distinguished from control based upon expression of the *CSF1R*-mApple reporter gene. The organs and tissues from the chicken CSF1-Fc-injected chickens visibly glowed red by comparison to the controls. Figure 8 shows *en face* views of skin and rectal mucosa, and a whole mount view of the cecum showing the cecal tonsil, of control and treated chicks. The view of the rectum also highlights the remarkable concentration of CSF1R^+^ macrophages in the lamina propria, as also seen in mice [12]. The conclusions based upon the whole mount view were supported in every tissue examined by confocal fluorescence microscopy (that is, spleen, bursa, liver, lung, kidney and muscle), where there was an apparent increase in the number of transgene-expressing cells. There also appeared to be an increase in the brightness of expression in individual cells (Additional file 2: Figure S7). In the spleen, the transgene expression corresponds to the known macrophage distribution in the white pulp, including inter-digitating dendritic cells and ellipsoid-associated cells, and in the red pulp [33]. Interestingly, the increase in fluorescent cells in the bursa of newly hatched chicks dosed with chicken CSF1-Fc was more striking in the macrophage population lining the connective tissue between the B cell follicles than in the population found in the medulla of the follicle, called bursal secretory dendritic cells. In the liver, the increased numbers of transgene-expressing Kupffer cells in response to chicken CSF1-Fc were clustered around sinusoids. A similar response was seen in the lung. Transgene-expressing cells were scattered throughout the interstitial tissue of the parabronchial wall in lung from a PBS-treated chick, the increased numbers of transgene-expressing cells in the chicken CSF1-Fc-treated chicks were not associated with a major change in distribution.

**Figure 9:**
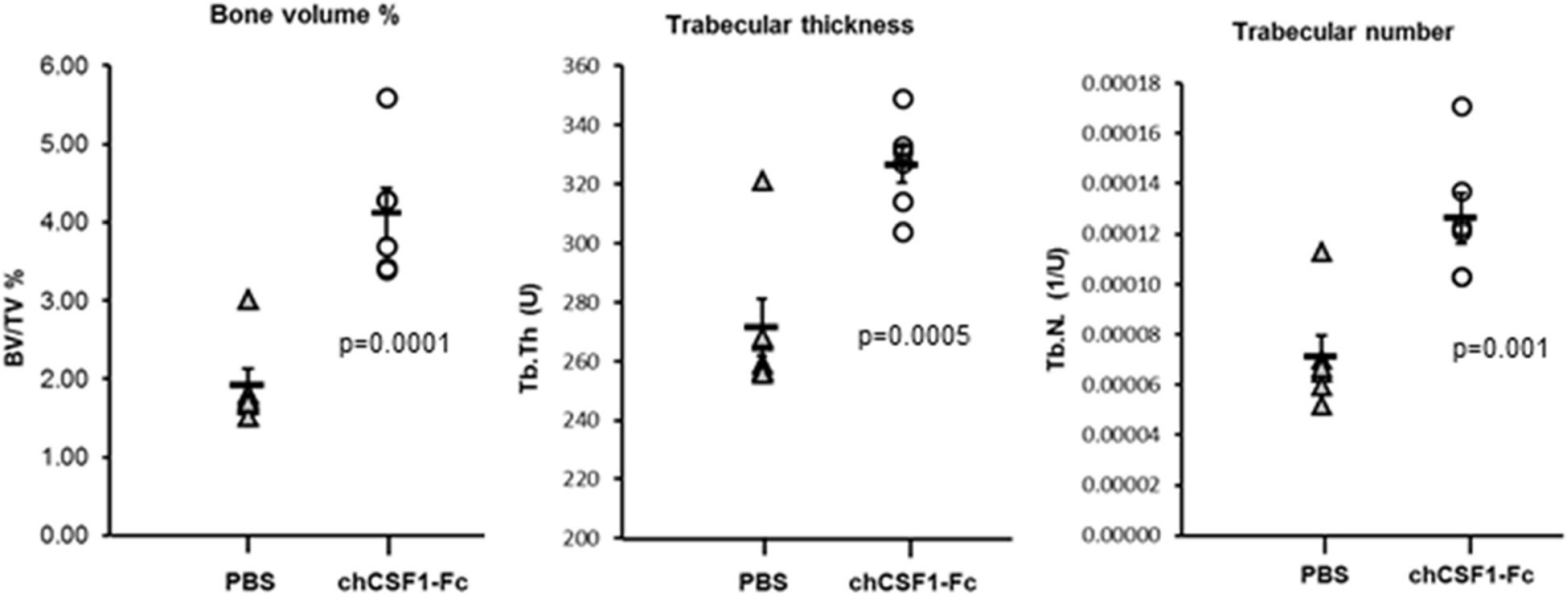
**The effect of chicken CSF1-Fc treatment on bone density.** Hatchlings were dosed subcutaneously with 50 μg chicken CSF1-Fc or PBS on hatch day (day 0), day 1, day 2 and day 3, then sacrificed 24 hours after the final injection. Femurs were fixed overnight in 4% paraformaldehyde, and then transferred to 70% ethanol. Micro-computed tomography analysis of the femur was carried out as described in Methods. Error bars represent standard error of the mean. Statistically significant differences were calculated using unpaired t-tests between treatment groups. PBS, phosphate-buffered saline.

Despite the apparent changes in tissue macrophages in the treated birds, there were no apparent adverse effects, at least in the short time they were examined. The treated and control groups gained weight at equal rates (data not shown). CSF1 treatment in mice was reported to cause splenomegaly and to promote liver and kidney growth or repair [34,35]. By contrast, there was no effect on any chicken organ weights in response to chicken CSF1-Fc. As demonstrated in the defective bone resorption of the *op/op* mouse, CSF1 has a crucial role in mammalian bone homeostasis through the differentiation of osteoclasts, the bone resorbing cells [36]. Conversely, the increased numbers of osteomacs and macrophages caused by the administration of CSF1 in a mouse tibial bone injury model was associated with increased matrix deposition and enhanced mineralization [37,38]. The femur structure of the PBS- and chicken CSF1-Fc-treated chicks was therefore analyzed by micro-computed tomography. Rather unexpectedly, the treatment increased bone formation. The bones of chicken CSF1-Fc-treated chicks actually displayed a two-fold higher bone volume to tissue volume than those of PBS-treated hatchlings. Furthermore, the trabecular thickness and the trabecular number were also increased in the chicken CSF1-Fc-treated femurs (Figure 9).

## Discussion

The yolk sac is believed to be the major source of progenitors of many mouse tissue macrophage populations, notably the microglia of the brain, based upon lineage trace studies [39]. In the present study, we have extended the previous evidence that the developing chicken contains an abundant macrophage population that can be detected by localization of *CSF1R* mRNA or novel *CSF1R* reporter genes [11,21]. The patterns of development, migration and localization detected using a *CSF1R* reporter were entirely consistent with previous descriptions of macrophage development in the mouse. Our previous generation of recombinant chicken CSF1 [11] permitted the production of large numbers of pure BMDM from chicken bone marrow. We compared their gene expression to the total gene expression in the embryo. The number of macrophages in the embryo was so high that it was possible with deep-RNAseq analysis to identify macrophage-expressed transcripts against the background of the mRNA from numerous other cell types. The chicken genome is still poorly assembled and annotated, and large parts of the sequence (especially some of the micro-chromosomes) are not even sequenced. Consequently, even after the *de novo* assembly of the tags that did not originally map to the genome, 10% of the new contigs were still not present in the current assembly. These contigs could form the basis of capture sequences to enable further refinement of the genome sequence. The primary sequence data have been provided to a consortium of avian researchers, and used to refine annotation of the chicken genome (Smith J, Burt D, *et al*., manuscript in preparation). As a first approximation, the data demonstrates that tissue macrophages in a developing chick have a similar transcriptome to CSF1-stimulated BMDM and, by inference, *CSF1R* mRNA and the *CSF1R* reporter expression accurately locates macrophage-like cells. A transcript annotated as the chemokine receptor *CCR2* is amongst the highly expressed genes shared by BMDM and embryonic macrophages in the chick. This gene is probably not a strict ortholog of mammalian *Ccr2* ([40], but the expression suggests a conserved function with the mammalian receptor.

The transplantation experiments described in this study demonstrate that bone marrow progenitors transplanted into a chick embryo before the onset of definitive hematopoiesis can produce long-lived and substantial chimerism restricted to the macrophage compartment. This observation will be technically useful for studies of the role of macrophages in innate immunity and the nature of immunological tolerance in birds. For example, one might anticipate transplanting marrow from birds that are resistance to a pathogen, such as *Salmonella* [41] or Marek’s disease virus [42], to determine whether resistance is macrophage-autonomous. The effectiveness of chimera generation, even without optimization, is especially surprising because we injected unfractionated bone marrow. There is clearly the potential to fractionate the marrow to identify *CSF1R*^+^ cells, and to modify the recipient genetically to reduce the contribution of endogenous hematopoietic progenitors to optimize this technology. The finding indicates that the early embryonic environment provides a niche that permits survival of clonogenic macrophage progenitors that can return to the marrow and repopulate the macrophage progenitor population, to produce a new generation of macrophages in response to CSF1. The same finding also indicates that the bone marrow contains a population of committed progenitor cells for the macrophage lineage that appear to be distinct from the pluripotent stem cells. Pluripotent stem cells did not apparently survive the embryonic environment available at the time of transfer, given that only macrophages were of donor origin in the chimeras. The macrophage progenitors that survive in the early embryonic environment may be the equivalent of the cells described recently in the mouse [43] or the high proliferative-potential macrophage colony-stimulating unit [44].

Yolk sac-derived blood cells that presented prior to the onset of definitive hematopoiesis were capable of extensive proliferation in response to chicken CSF1 *ex vivo*. When transplanted, they were apparently able to populate the same macrophage populations in the embryo as those derived from the bone marrow. But, unlike bone marrow-derived cells, macrophages derived from yolk sac donors were not retained into the adult bird (Figure 3). Because neither the yolk sac cells, nor the bone marrow cells, were pure populations, we cannot eliminate the possibility that there was a quantitative difference in the number of transferred precursor cells. The recent literature from mouse systems, based on the study of lineage trace and myb-knockout mice, has accepted that many yolk sac-derived macrophages persist as a substantial distinct population in the adult [45]. By contrast, a recent study by Epelman *et al*. [46] concluded that liver, heart and brain were the only mouse organs in which yolk sac-derived macrophages persist into adulthood in significant numbers, and even then they were a minor subpopulation. As discussed elsewhere [2,3], our interpretation of the data in mouse and the current data for the chick is that yolk sac-derived and definitive macrophages can occupy the same specific niches and in normal development the cells derived from the yolk sac are replaced by fetal liver or bone marrow monocyte-derived cells.

We have shown that CSF1 and IL34 are expressed during chicken embryonic development. By injecting a novel form of recombinant CSF1, chicken CSF1-Fc, into both the developing embryo and the hatchling bird, we have shown that CSF1R signaling is limiting for the proliferation and differentiation of tissue macrophages in birds as it is in mammals. *Inter alia*, these observations further highlight the utility of the MacGreen and MacRed reporter genes in the chick to monitor alterations in mononuclear phagocyte number, location and behavior [21]. One unexpected effect of chicken CSF1-Fc in hatchlings was the increase in lymphocytes as well as monocytes, since these cells do not express CSF1R. This could arise because of the remarkable concentration of CSF1R-expressing cells in white pulp of spleen, and in the bursa. This is not the case in mammals, where macrophages are largely confined to red pulp and marginal zones. It may be that the CSF1-stimulated macrophages produce factors that promote lymphocyte proliferation or migration. Alternatively, in the mouse, pigCSF1-Fc treatment greatly increased tissue macrophage numbers, through both local proliferation and monocyte extravasation [47]. So, in the treated chicks, the chicken CSF1-Fc could act in at least two ways, by promoting proliferation of precursors and tissue macrophages, and by driving the monocytes selectively to leave the circulation.

## Conclusion

Data we have presented here support the use of the chick model in the study of monocytopoiesis, and demonstrate that CSF1R transgenic reporters will enable the visualization of key events in monocyte-macrophage development.

## Methods

### Antibodies

Antibodies and dilutions used were as follows: chicken CD3 (clone CT3, Southern Biotech, Birmingham, Alabama, USA), 1:100 for FACS; Bu-1 (clone AV20, Southern Biotech), 1:1,000 for FACS, 1:300 for immunofluorescence; CD45 (clone AV53), 1:1,000 for FACS and immunofluorescence; chicken CSF1R [20], 1:100 for immunofluorescence; GFP Rabbit IgG Antibody Fraction, Alexa Fluor 488 Conjugate (Invitrogen, Carlsbad, CA, USA), 1:500 for immunofluorescence; Goat-anti-mouse-IgG1-alexa 647 (Invitrogen), 1:5,000 for FACS; Alexa Fluor 546 F(ab')2 Fragment of Goat Anti-Mouse IgG (Invitrogen), 1:500 for immunofluorescence.

### Cells and animals

DF-1 cells were originally obtained from the American Type Culture Collection (ATCC, UK), and grown in Dulbecco’s modified minimal essential medium (DMEM) supplemented with 100 μg/ml penicillin and 100 μg/ml streptomycin (P/S), 10% FBS (PAA/GE Healthcare Life Sciences, UK) and 2% chick serum (Sigma-Aldrich, UK) by volume at 39°C.

CHO cells were originally obtained from ATCC, and grown in Hams-F12 media (Life Technologies, UK) supplemented with 10% FBS, non-essential amino acids and sodium pyruvate at 37°C in 5% CO_2._

Ba/F3 cells expressing the chicken CSF1R were described previously [11], and were used in the generation and characterization of monoclonal antibodies against the chicken CSF1R [20], also used herein. They were maintained in RPMI-1640 medium (Sigma-Aldrich) supplemented with 10% heat-inactivated FBS, 2 mM L-glutamine and antibiotics (100 g/ml penicillin, 100 g/ml streptomycin) and 10% conditioned medium from X63 Ag8-653 myeloma cells carrying an expression vector for IL-3 [48] at 37°C.

Chicken bone marrow cells were obtained from day-old chicks by flushing the marrow from femurs and tibias with PBS using a syringe and a blunt needle. The marrow from multiple bones was pooled and washed once in RPMI-1640, passed through a 100 μm cell strainer (Falcon), and resuspended in a small volume (150 μl per bone) for micro-injection. BMDM were produced by adapting methods previously employed for mouse and pig cells in our laboratory [49]. Briefly, marrow cells were harvested from femurs of hatchling birds, resuspended in RPMI supplemented with 10% heat-inactivated FCS, 2 mM L-glutamine, 100 μg/ml penicillin, 100 μg/ml streptomycin, and 125 ng/ml recombinant chicken CSF1 ([11] and plated in 60 mm bacteriological plates (Sterilin) at a density equivalent to one bone per plate. Cells were then incubated at 37°C in a CO_2_ incubator for 7 days.

Chicken circulating yolk sac-derived cells were obtained by extracting blood from the dorsal aorta of stage HH16 to HH17 embryos with ubiquitous EGFP expression using a fine glass micropipette under a stereomicroscope. Blood from five embryos was pooled and 20 μl of whole blood added to 2 ml of medium in six-well Corning Costar cell culture plates. Medium was composed of DMEM High glucose - GlutaMAX (Life Technologies), supplemented with 10% heat-inactivated FCS, 10% Gibco MEM Non-Essential Amino Acids, 100 μg/ml penicillin, 100 μg/ml streptomycin, and in some cases 350 ng/ml recombinant chicken CSF1 at 37°C.

For zymosan phagocytosis and confocal analysis after 10 days in culture with CSF1, macrophages were transferred into four-well Nunc Lab-Tek Chamber Slides and cultured for a further 48 hours. In some cases, 100 μg/ml of Texas Red-conjugated zymosan BioParticles (Life Technologies) was added to cells for one hour, then washed four times and fresh medium was added to cells for 24 hours. Cells were fixed for 30 minutes at room temperature with 4% PFA/PBS and washed three times with PBS. Cells were stained as described below.

Wild-type ISA Brown layer birds, and constitutive EGFP-expressing birds derived from the same genetic background [18] were obtained from the National Avian Research Facility at The Roslin Institute, Edinburgh. Transgenic birds expressing either mApple or EGFP driven by promoter and enhancer elements of the chicken CSF1R locus are described in detail elsewhere [21]. These birds were also maintained at the National Avian Research Facility. Young chick embryos were staged in accordance with Hamilton and Hamburger’s tables [50]. The ages of more advanced embryos and hatched chicks are indicated by the incubation or post-hatching day.

### RNAseq

RNA from chicken BMDM (produced as described above), day 7 embryos or DF-1 cells was extracted using TRIzol reagent, as described by the manufacturers (Invitrogen). RNA was quantified using a Nanodrop and RNA quality was assessed using an Agilent Nano Chip kit. Sequencing was performed by Ark Genomics (Roslin Institute) using the Illumina HiSeq 2000 Sequencing System. Short reads were filtered for quality, and mapped to the annotated chicken genome using TopHat. Read counts production, normalization of data, gene annotation and *de novo* assembly of the unmapped reads were performed by Edinburgh Genomics staff within The Roslin Institute. Enriched candidate genes were determined by calculating the ratio of the read counts between chicken BMDM and DF-1, embryos and DF-1, or chicken BMDM and embryos. RNAseq data have been lodged with Array Express (E-MTAB-3048) and contributed to the chicken RNAseq Consortium.

### *In situ* hybridization

Fertilized White Leghorn eggs were collected weekly and incubated at 38°C for between 3 and 6 days of development. Embryos were dissected into cold DEPC-PBS, staged as above, fixed immediately in 4% PFA with DEPC-PBS overnight, dehydrated into 100% methanol through gradual methanol and PBS steps, and stored at -20°C. The CSF1R and IL34 probes were made using cDNA clones 654 and 591, respectively, obtained from the Edinburgh Genomics (formerly ARK Genomics, Roslin, UK) as the template. Whole-mount *in situ* hybridization on embryos was carried out as described [51].

### Chicken CSF1-Fc production

We have described elsewhere the production of fusion protein between pig CSF1 and the Fc fragment of immunoglobulin [31]. To produce a similar form of chicken CSF1, we fused the coding sequence of the minimal active fragment of chicken CSF1 (MPRLGSQVSLFRCTLLSSLLLVCSIHETEQNSYCQQIITERHLDHLQELADTQMQQPGTVSFRFISKMRLSDSVCYVKAAFPLLGTILNRTTFKENSTNANKMKTVRKMYENIDENVDPCIRDEDDKEHALSEMCFEEFTTSPYEMLVLVRQFFQDIKQLLQNKETFEKDCSQVYRSACAGPRQHSSSP) with a linker (GGGGS), the chicken IgY constant heavy chain domains 3 and 4 (DGAQSCSPIQLYAIPPSPGELYISLDAKLRCLVVNLPSDSSLSVTWTREKSGNLRPDPMVLQEHFNGTYSASSAVPVSTQDWLSGERFTCTVQHEELPLPLSKSVYRNTGPTTPPLIYPFAPHPEELSLSRVTLSCLVRGFRPRDIEIRWLRDHRAVPATEFVTTAVLPEERTANGAGGDGDTFFVYSKMSVETAKWNGGTVFACMAVHEALPMRFSQRTLQKQAGK) and a histidine tag (HHHHHH). The entire amino acid sequence was reverse-translated and codon-optimized for expression Chinese Hamster Ovary (CHO) and synthesized by Blue Heron Biotechnologies (WA, USA). The sequence was engineered with a HindIII restriction site and Kozak sequence (GCCACC) at the 5′ end, and a stop codon and EcoRI restriction site at the 3′ end, and cloned into the expression plasmid pCDNA3.1(+) (Invitrogen) using the complementary restriction sites. The resulting plasmid was subsequently used as the source of DNA for subcloning into a stable cell expression system.

One day before transfection, 5 × 10^6^ cells CHO cells were seeded in 6 ml growth medium without antibiotics in a T25 flask, and 24 hours later were transfected with 10 μg cCSF1R-Ig + pFUSE-IgG1-Fc DNA using Lipofectamine 2000 (Life Technologies), following manufacturer’s instructions. The following day, the transfection medium was replaced with growth medium supplemented with 250 μg/ml Zeocin (InvivoGen, Toulouse, France) to select for Zeocin-resistant transfectants. After 3 weeks of growth in selective medium, foci of Zeocin-resistant stable transfectants were screened, cloned and expanded.

The Fc fusion protein was isolated using a combination of mixed modal and immobilized metal ion affinity chromatography steps. Conditioned medium from the transfected CHO cell culture was clarified and sodium citrate was added to a 50 mM final concentration. The pH of the conditioned medium was adjusted to 5.5 and the medium was clarified by filtration. Chromatography was performed on Capto MMC. The GE was as follows: the resin was equilibrated with 50 mM sodium citrate pH 5.5 (A1), the medium was loaded and the column was washed with A1 until the A280 returned to baseline. The resin was washed with 50 mM sodium citrate pH 5.5, 0.7 M NaCl (A2) and a gradient to 50 mM Tris pH 8.5; 0.5 M NaCl was used to elute the fusion protein-rich fraction. Affinity chromatography was accomplished using Ni-NTA resin, equilibrated with A buffer (50 mM Tris pH 8, 300 mM NaCl). The Capto MMC-rich pool was batch-bound to resin for 60 minutes and elution of the desired protein was performed with an imidazole gradient. Protein-rich fractions were pooled based on SDS-PAGE and dialyzed into PBS.

### Cell transplantation and embryo culture

Blood was extracted from the dorsal aorta of stage HH16 to HH17 embryos with ubiquitous EGFP expression using a fine glass micropipette under a stereomicroscope. Approximately 5 to 7 μl of blood was collected per embryo and the blood from 10 embryos was pooled. Bone marrow cells from day-old chicks were extracted as described above. Yolk sac hematopoietic or bone marrow cell chimeras were made by microinjecting a 3 to 5 μl aliquot of EGFP^+^ blood or bone marrow into the dorsal aorta of aged-matched non-transgenic recipient embryos. Recipient embryos were monitored throughout embryonic development to assess transplantation of EGFP^+^ cells. To generate adult yolk sac hematopoietic or bone marrow chimeras, embryos were transferred into phase III host shells and cultured to hatching as described [18].

### Flow cytometric analysis

Heparinized blood was diluted with PBS to about 3 ml and layered on top of 3 ml Histopaque 1.077 in a 15 ml Falcon tube, and cells isolated by centrifugation at 400 × g for 20 min and both acceleration and deceleration set at 1. Cells at the interface were washed three times with PBS, spun at 350 × g for 5 minutes at 4°C, and resuspended in 0.5 ml FACS buffer (PBS supplemented with 0.5% BSA and 0.05% NaN3). Tissues were collected in ice-cold PBS, finely chopped with microscissors and passed through 100 μm cell strainers (Falcon). Chicken bone marrow cells were washed three times with PBS (350 × g for 5 minutes at 4°C), passed through a 100 μm cell strainer, and 10^6^ cells added per well in a U-shaped 96-well plate. Cells were stained sequentially with primary and secondary antibody, washed three times in buffer between incubations by centrifugation for 3 minutes at 350 × g and 4°C. Cells were resuspended in FACS buffer (100 μl/well), and transferred to FACS tubes filled with 200 μl FACS buffer. Analysis was performed on a FACScalibur flowcytometer (BD Biosciences, UK). Data were analyzed using the software program Summit v4.3 (Dako, UK).

### Chicken CSF1-Fc microinjection in embryos and whole mount fluorescence imaging

MacGreen fertile eggs were incubated at 38°C for 4.5 days prior to injection. Micro-capillary tubes (Harvard Apparatus, UK) with a 1.5 mm external and 1.17 mm internal diameter were made into glass needles using a moving-coil microelectrode puller, model 753 (Campden Instruments Ltd, UK). Sharp scissors were used to make a hole in the shell of an egg at the blunt end. The embryo was visualized by cutting a window in the eggshell. Chicken CSF1-Fc conjugate was taken up into a glass capillary and injected in the neural tube of the host embryos. Control embryos were injected with PBS vehicle. Windowed eggs were resealed with tape and returned to the incubator for 36 hours. Embryos were removed from the egg, placed in PBS, and whole mount imaging performed using a Leica MZFLIII fluorescent microscope.

### Embryo fixation and confocal fluorescence imaging

Embryos were fixed for 1 hour in 4% paraformaldehyde in PBS, washed in PBS and incubated overnight in 18% sucrose in PBS. Embryos were then cryo-embedded in Tissue-Tek OCT compound (Sakura Finetechnical, Tokyo, Japan) and sectioned at 10 μm onto SuperFrost Plus slides (Menzel-Gläser, Germany). Sections were left to dry, and visualized using an inverted confocal microscope (Nikon eC1; Nikon Instruments). Images were captured using Nikon EZ- C1 Software v3.40. Lymphoid tissues in embryonic and post-hatch birds were imaged by dissecting the relevant organ, which was placed in a petri-dish for whole mount imaging using a Leica MZFLIII fluorescent microscope. Tissues were fixed and embedded as above. Sections were left to dry, then blocked for 1 hour in 10% skim milk powder, 10% normal horse sera and 0.1% Triton X-100 in PBS (MST- PBS). Primary antibodies were added (anti-CSF1R, anti-CD45, anti-Bu-1), diluted in MST-PBS and sections incubated at 4°C overnight. Sections then were washed for 30 minutes in PBS and re-incubated with secondary antibody (Alexa Fluor 546 F(ab')2 Fragment of Goat Anti-Mouse IgG (Invitrogen) and anti-GFP Rabbit IgG Antibody Fraction, Alexa Fluor® 488 Conjugate (Invitrogen)) diluted in MST-PBS for one hour. Sections were washed for 30 minutes in PBS, and mounted in Hydromount (National Diagnostics, Atlanta, Georgia, USA). Cells were imaged using an inverted confocal microscope (Nikon eC1; Nikon Instruments).

### Subcutaneous injection of chicken CSF1-Fc in hatchlings

Freshly hatched MacRed chicks were weighed and divided into treatment groups that were matched for body weight. Six MacRed and two wild-type chicks were sacrificed prior to the course of injection for baseline level assessment. Subcutaneous injections were performed at the same time daily, at two different sites in the upper body with 1 mg/kg (50 μg chicken CSF1- Fc/chick in PBS) for four treatments (day 0 to day 3). Control birds were injected with PBS. Birds were weighed daily, prior to injection, and on the day of sacrifice (day 4). Chicks were sacrificed 24 hours post-last injection. Blood was collected using heparinized syringes and needles. Whole mount tissues were imaged under UV light using a red fluorescence filter for the same exposure time. All tissues were then fixed in 4% PFA for 4 hours followed by overnight incubation at 4°C in 18% sucrose, except one lobe of each liver, one piece of muscle of every chick, and every other small intestine (whole), which were put in RNAlater (QIAGEN) and stored at -80°C. Tibias were kept in cold PBS until bone marrow extraction for FACS analysis of transgene expression. Femurs were fixed in 4% PFA, and sent for micro computed tomography using a Skyscan 1172 scanner (Skyscan, Kontich, Belgium) and sectioning. PFA-fixed tissues were cryo-embedded in Tissue-Tek OCT compound (Sakura Finetechnical) and sectioned at 10 μm onto SuperFrost Plus slides (Menzel-Gläser). Sections were left to dry and visualized using an inverted confocal microscope (Nikon eC1; Nikon Instruments). Images were captured using Nikon EZ-C1 Software v3.40.

## Additional files

Additional file 1: Table S1. Transcription factors known to be involved in myeloid lineage-specific hematopoiesis. **Table S2.** Known macrophage-expressed genes that were detected at comparable levels to *CSF1R* in total embryo mRNA. **Table S3.** Genes with enriched expression in chicken BMDM relative to fibroblasts but not in embryonic macrophages. Additional file 2: Figure S1. Generation of bone marrow chimeras. **Figure S2.** EGFP-expressing cells in the brain parenchyma of chimeras. (A) Co-localization of EGFP and CSF1R. (B) Co-localization of EGFP and CD45. Scale bars = 500 μM. **Figure S3A.** Characterization of chimeric spleen. (A) Representative FACS plot. (B) Representative fluorescence profiles for large granular EGFP^+^ cells in control. (C) Chimerism depends upon gates, conservative suggests 45% (green), and relaxed (blue) suggests 55%. (D) Chimerism in three birds, based upon conservative gating. (E) No co-expression of EGFP and B cell marker Bu-1 (red) (upper panel). Co-expression of EGFP and CSF1R (red) (lower panel). **Figure S3B.** Characterization of chimeric bursa of Fabricius. (A) Representative FACS plot. Cell populations were divided according to size and granularity, and number of EGFP^+^ cells determined. (B) No co-expression of EGFP and Bu-1 (red) (left panel). Co-expression of EGFP and CSF1R (red) (right panel). Scale bars = 100 μM. **Figure S4.** (A) Localization of *IL34* mRNA by whole mount *in situ*. Sense control probe (left). Antisense probe (right). Upper panel: Arrows indicate regions of stronger expression. Lower panel: The head. Arrow indicates the notochord. (B) Expression in the embryo’s head by quantitative RT-PCR. **Figure S5.** Relative bioactivity of chicken CSF1 and chicken CSF1-Fc on chicken CSF1R-expressing Ba/F3. **Figure S6.** Fertile MacGreen eggs injected with PBS (left) or chicken CSF1-Fc (right). (A) Upper panels were under visible light (12.5×), lower panels show hindlimbs (hl) under UV light using a GFP filter (40×). (B) Upper panels show 10 μm sagittal section through the somites. Lower panels show the hindlimb. Scale bars = 200 μM. **Figure S7.** Transgene-expressing cells in tissues from MacRed chicks treated with chicken CSF1-Fc. Spleen (i); bursa of Fabricius (ii); liver (iii); lung (iv); kidney (v); brain (vi); muscle, cross (vii); muscle, longitudinal (viii). Scale bars: A,B,E,F,G,H = 50 μM; C = 25 μM; D = 100 μM.

## References

[1] Hume DA (2006). The mononuclear phagocyte system. Curr Opin Immunol..

[2] Gentek R, Molawi K, Sieweke MH (2014). Tissue macrophage identity and self-renewal. Immunol Rev..

[3] Jenkins SJ, Hume DA (2014). Homeostasis in the mononuclear phagocyte system. Trends Immunol..

[4] Cuadros MA, Martin C, Coltey P, Almendros A, Navascues J (1993). First appearance, distribution, and origin of macrophages in the early development of the avian central nervous system. J Comp Neurol..

[5] Cuadros MA, Coltey P, Carmen Nieto M, Martin C (1992). Demonstration of a phagocytic cell system belonging to the hemopoietic lineage and originating from the yolk sac in the early avian embryo. Development..

[6] Lichanska AM, Browne CM, Henkel GW, Murphy KM, Ostrowski MC, McKercher SR (1999). Differentiation of the mononuclear phagocyte system during mouse embryogenesis: the role of transcription factor PU.1. Blood..

[7] Lichanska AM, Hume DA (2000). Origins and functions of phagocytes in the embryo. Exp Hematol..

[8] Naito M, Yamamura F, Nishikawa S, Takahashi K (1989). Development, differentiation, and maturation of fetal mouse yolk sac macrophages in cultures. J Leukoc Biol..

[9] Hume DA, MacDonald KP (2012). Therapeutic applications of macrophage colony-stimulating factor-1 (CSF-1) and antagonists of CSF-1 receptor (CSF-1R) signaling. Blood..

[10] Nakamichi Y, Udagawa N, Takahashi N (2013). IL-34 and CSF-1: similarities and differences. J Bone Miner Metab..

[11] Garceau V, Smith J, Paton IR, Davey M, Fares MA, Sester DP (2010). Pivotal advance: Avian colony-stimulating factor 1 (CSF-1), interleukin-34 (IL-34), and CSF-1 receptor genes and gene products. J Leukoc Biol..

[12] Sasmono RT, Oceandy D, Pollard JW, Tong W, Pavli P, Wainwright BJ (2003). A macrophage colony-stimulating factor receptor-green fluorescent protein transgene is expressed throughout the mononuclear phagocyte system of the mouse. Blood..

[13] Schulz C, Gomez Perdiguero E, Chorro L, Szabo-Rogers H, Cagnard N, Kierdorf K (2012). A lineage of myeloid cells independent of Myb and hematopoietic stem cells. Science..

[14] Dai X-M, Ryan GR, Hapel AJ, Dominguez MG, Russell RG, Kapp S (2002). Targeted disruption of the mouse colony-stimulating factor 1 receptor gene results in osteopetrosis, mononuclear phagocyte deficiency, increased primitive progenitor cell frequencies, and reproductive defects. Blood..

[15] Erblich B, Zhu L, Etgen AM, Dobrenis K, Pollard JW (2011). Absence of colony stimulation factor-1 receptor results in loss of microglia, disrupted brain development and olfactory deficits. PLOS One..

[16] Ginhoux F, Greter M, Leboeuf M, Nandi S, See P, Gokhan S (2010). Fate mapping analysis reveals that adult microglia derive from primitive macrophages. Science..

[17] Hashimoto D, Chow A, Noizat C, Teo P, Beasley MB, Leboeuf M (2013). Tissue-resident macrophages self-maintain locally throughout adult life with minimal contribution from circulating monocytes. Immunity..

[18] McGrew MJ, Sherman A, Lillico SG, Ellard FM, Radcliffe PA, Gilhooley HJ (2008). Localised axial progenitor cell populations in the avian tail bud are not committed to a posterior Hox identity. Development..

[19] Zhao D, McBride D, Nandi S, McQueen HA, McGrew MJ, Hocking PM (2010). Somatic sex identity is cell autonomous in the chicken. Nature..

[20] Garcia-Morales C, Rothwell L, Moffat L, Garceau V, Balic A, Sang HM (2014). Production and characterisation of a monoclonal antibody that recognises the chicken CSF1 receptor and confirms that expression is restricted to macrophage-lineage cells. Dev Comp Immunol..

[21] Balic A, Garcia-Morales C, Vervelde L, Gilhooley H, Sherman A, Garceau V (2014). Visualisation of the avian mononuclear phagocyte system using novel transgenic reporter genes based upon conserved elements of the CSF1R locus. Development..

[22] Freeman TC, Ivens A, Baillie JK, Beraldi D, Barnett MW, Dorward D (2012). A gene expression atlas of the domestic pig. BMC Biol..

[23] Hume DA, Summers KM, Raza S, Baillie JK, Freeman TC (2010). Functional clustering and lineage markers: insights into cellular differentiation and gene function from large-scale microarray studies of purified primary cell populations. Genomics..

[24] Mabbott NA, Kenneth Baillie J, Hume DA, Freeman TC (2010). Meta-analysis of lineage-specific gene expression signatures in mouse leukocyte populations. Immunobiology..

[25] Jaffredo T, Gautier R, Eichmann A, Dieterlen-Lievre F (1998). Intraaortic hemopoietic cells are derived from endothelial cells during ontogeny. Development..

[26] Saynajakangas R, Uchida T, Vainio O (2009). Differential gene expression in CD45 cells at para-aortic foci stage of chicken haematopoiesis. Scand J Immunol..

[27] Macdonald J, Taylor L, Sherman A, Kawakami K, Takahashi Y, Sang HM (2012). Efficient genetic modification and germ-line transmission of primordial germ cells using piggyBac and Tol2 transposons. Proc Natl Acad Sci U S A..

[28] Wong GK, Cavey MJ (1993). Development of the liver in the chicken embryo. II. Erythropoietic and granulopoietic cells. Anat Rec.

[29] Le Douarin NM, Dieterlen-Lievre F, Oliver PD (1984). Ontogeny of primary lymphoid organs and lymphoid stem cells. Am J Anat..

[30] Houssaint E, Lassila O, Vainio O (1989). Bu-1 antigen expression as a marker for B cell precursors in chicken embryos. Eur J Immunol..

[31] Gow DJ, Sauter KA, Pridans C, Moffat L, Sehgal A, Stutchfield BM (2014). Characterisation of a novel Fc conjugate of macrophage colony-stimulating factor. Mol Ther..

[32] Ulich TR, del Castillo J, Watson LR, Yin SM, Garnick MB (1990). In vivo hematologic effects of recombinant human macrophage colony-stimulating factor. Blood..

[33] Jeurissen SH, Janse EM (1989). Distribution and function of non-lymphoid cells in liver and spleen of embryonic and adult chickens. Prog Clin Biol Res..

[34] Alikhan MA, Jones CV, Williams TM, Beckhouse AG, Fletcher AL, Kett MM (2011). Colony-stimulating factor-1 promotes kidney growth and repair via alteration of macrophage responses. Am J Pathol..

[35] Hume DA, Pavli P, Donahue RE, Fidler IJ (1988). The effect of human recombinant macrophage colony-stimulating factor (CSF-1) on the murine mononuclear phagocyte system in vivo. J Immunol..

[36] Felix R, Hofstetter W, Wetterwald A, Cecchini MG, Fleisch H (1994). Role of colony-stimulating factor-1 in bone metabolism. J Cell Biochem..

[37] Alexander KA, Chang MK, Maylin ER, Kohler T, Muller R, Wu AC (2011). Osteal macrophages promote in vivo intramembranous bone healing in a mouse tibial injury model. J Bone Miner Res..

[38] Chang MK, Raggatt LJ, Alexander KA, Kuliwaba JS, Fazzalari NL, Schroder K (2008). Osteal tissue macrophages are intercalated throughout human and mouse bone lining tissues and regulate osteoblast function in vitro and in vivo. J Immunol..

[39] Epelman S, Lavine KJ, Randolph GJ (2014). Origin and functions of tissue macrophages. Immunity..

[40] Hughes S, Poh TY, Bumstead N, Kaiser P (2007). Re-evaluation of the chicken MIP family of chemokines and their receptors suggests that CCL5 is the prototypic MIP family chemokine, and that different species have developed different repertoires of both the CC chemokines and their receptors. Dev Comp Immunol..

[41] Fife MS, Howell JS, Salmon N, Hocking PM, van Diemen PM, Jones MA (2010). Genome-wide SNP analysis identifies major QTL for Salmonella colonization in the chicken. Anim Genet..

[42] Smith J, Sadeyen JR, Paton IR, Hocking PM, Salmon N, Fife M (2011). Systems analysis of immune responses in Marek’s disease virus-infected chickens identifies a gene involved in susceptibility and highlights a possible novel pathogenicity mechanism. J Virol..

[43] Hettinger J, Richards DM, Hansson J, Barra MM, Joschko AC, Krijgsveld J (2013). Origin of monocytes and macrophages in a committed progenitor. Nat Immunol..

[44] Breen FN, Hume DA, Weidemann MJ (1991). Interactions among granulocyte-macrophage colony-stimulating factor, macrophage colony-stimulating factor, and IFN-gamma lead to enhanced proliferation of murine macrophage progenitor cells. J Immunol..

[45] Lavin Y, Merad M (2013). Macrophages: gatekeepers of tissue integrity. Cancer Immunol Res..

[46] Epelman S, Lavine KJ, Beaudin AE, Sojka DK, Carrero JA, Calderon B (2014). Embryonic and adult-derived resident cardiac macrophages are maintained through distinct mechanisms at steady state and during inflammation. Immunity..

[47] Jenkins SJ, Ruckerl D, Thomas GD, Hewitson JP, Duncan S, Brombacher F (2013). IL-4 directly signals tissue resident macrophages to proliferate beyond homeostatic levels controlled by CSF-1. J Exp Med..

[48] Karasuyama H (1988). Establishment of mouse cell lines which constitutively secrete large quantities of interleukin 2, 3, 4 or 5, using high-copy cDNA expression vectors. Tanpakushitsu Kakusan Koso.

[49] Kapetanovic R, Fairbairn L, Beraldi D, Sester DP, Archibald AL, Tuggle CK (2012). Pig bone marrow-derived macrophages resemble human macrophages in their response to bacterial lipopolysaccharide. J Immunol..

[50] Hamburger V, Hamilton HL. A series of normal stages in the development of the chick embryo. Dev Dyn. 1992. 1951;195:231–72. 10.1002/aja.1001950404 1304821

[51] Nieto MA, Patel K, Wilkinson DG (1996). In situ hybridization analysis of chick embryos in whole mount and tissue sections. Methods Cell Biol..

