# Supplementary material for: The development and maintenance of the mononuclear phagocyte system of the chick is controlled by signals from the macrophage colony-stimulating factor receptor
Source: BMC Biol. 2015 Feb 19;13:12. doi: 10.1186/s12915-015-0121-9 (PMC4369834; doi:10.1186/s12915-015-0121-9)

**Additional file 1: Tables**

**Additional file 1: Table S1. Transcription factors known to be involved in myeloid lineage-specific haematopoiesis.**


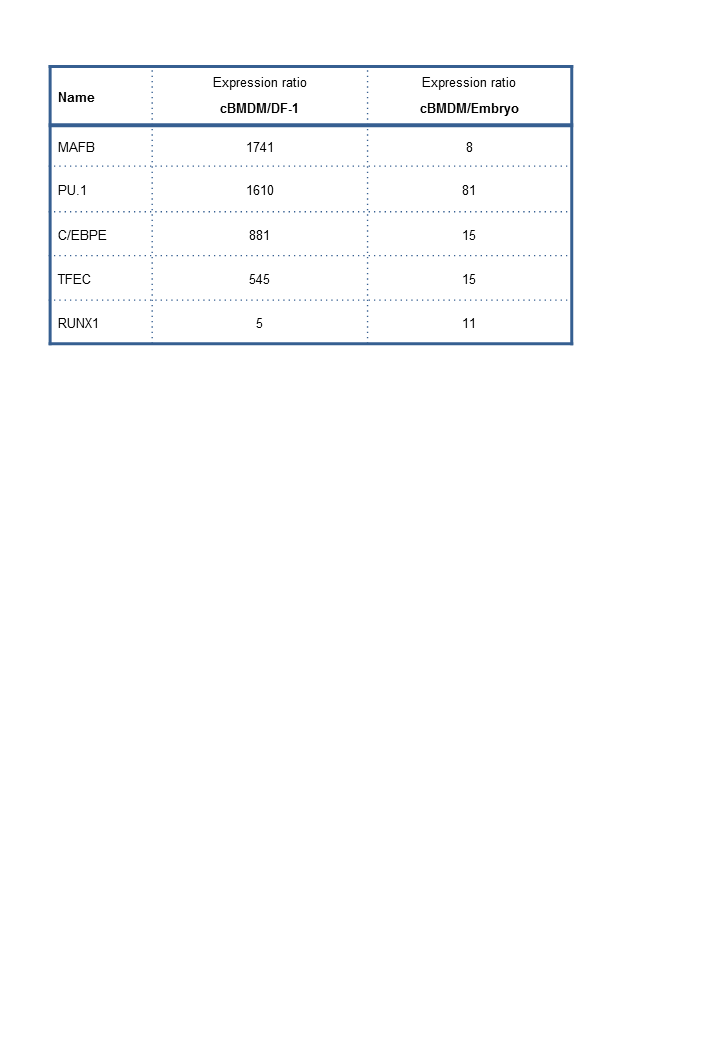


**Additional file 1: Table S2. Known macrophage-expressed genes that were detected at comparable levels to Csf1r in total embryo mRNA.**


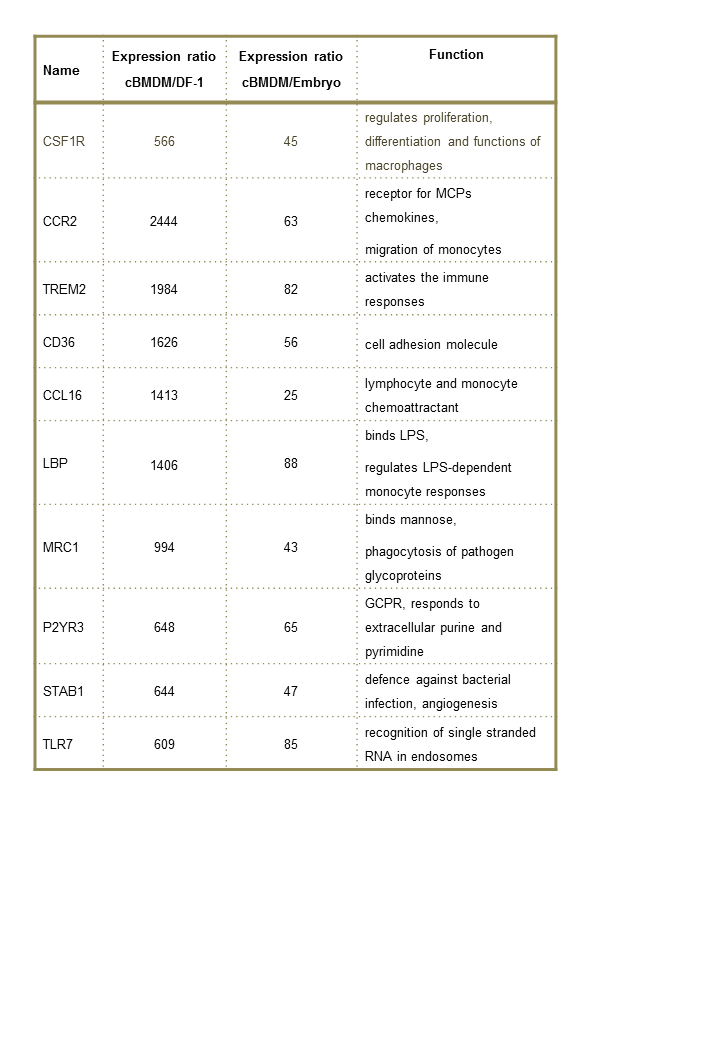


**Additional file 1: Table S3** Genes with enriched expression in chicken BMDM relative to fibroblasts but not in embryonic macrophages.


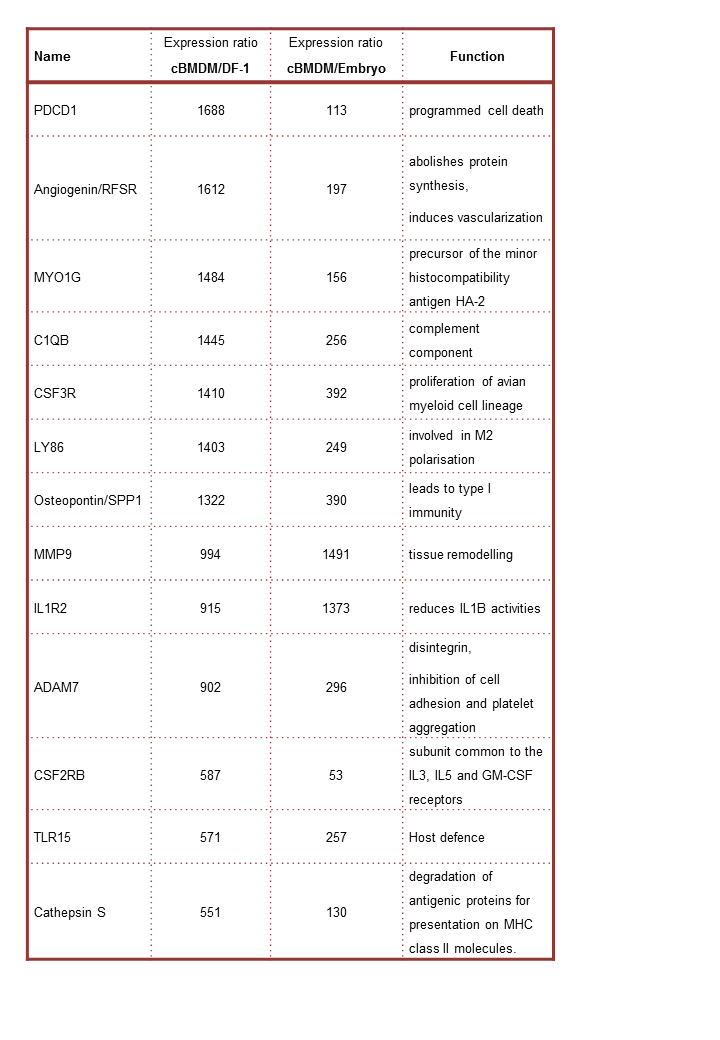

Supplement: Additional file 1: Table S1. — Transcription factors known to be involved in myeloid lineage-specific hematopoiesis. Table S2. Known macrophage-expressed genes that were detected at comparable levels to CSF1R in total embryo mRNA. Table S3. Genes with enriched expression in chicken BMDM relative to fibroblasts but not in embryonic macrophages. [file 12915_2015_121_MOESM1_ESM.docx]
