# Supplementary material for: The development and maintenance of the mononuclear phagocyte system of the chick is controlled by signals from the macrophage colony-stimulating factor receptor
Source: BMC Biol. 2015 Feb 19;13:12. doi: 10.1186/s12915-015-0121-9 (PMC4369834; doi:10.1186/s12915-015-0121-9)
Supplement: Additional file 2: Figure S1. — Generation of bone marrow chimeras. Figure S2. EGFP-expressing cells in the brain parenchyma of chimeras. (A) Co-localization of EGFP and CSF1R. (B) Co-localization of EGFP and CD45. Scale bars = 500 μM. Figure S3A. Characterization of chimeric spleen. (A) Representative FACS plot. (B) Representative fluorescence profiles for large granular EGFP+ cells in control. (C) Chimerism depends upon gates, conservative suggests 45% (green), and relaxed (blue) suggests 55%. (D) Chimerism in three birds, based upon conservative gating. (E) No co-expression of EGFP and B cell marker Bu-1 (red) (upper panel). Co-expression of EGFP and CSF1R (red) (lower panel). Figure S3B. Characterization of chimeric bursa of Fabricius. (A) Representative FACS plot. Cell populations were divided according to size and granularity, and number of EGFP+ cells determined. (B) No co-expression of EGFP and Bu-1 (red) (left panel). Co-expression of EGFP and CSF1R (red) (right panel). Scale bars = 100 μM. Figure S4. (A) Localization of IL34 mRNA by whole mount in situ. Sense control probe (left). Antisense probe (right). Upper panel: Arrows indicate regions of stronger expression. Lower panel: The head. Arrow indicates the notochord. (B) Expression in the embryo’s head by quantitative RT-PCR. Figure S5. Relative bioactivity of chicken CSF1 and chicken CSF1-Fc on chicken CSF1R-expressing Ba/F3. Figure S6. Fertile MacGreen eggs injected with PBS (left) or chicken CSF1-Fc (right). (A) Upper panels were under visible light (12.5×), lower panels show hindlimbs (hl) under UV light using a GFP filter (40×). (B) Upper panels show 10 μm sagittal section through the somites. Lower panels show the hindlimb. Scale bars = 200 μM. Figure S7. Transgene-expressing cells in tissues from MacRed chicks treated with chicken CSF1-Fc. Spleen (i); bursa of Fabricius (ii); liver (iii); lung (iv); kidney (v); brain (vi); muscle, cross (vii); muscle, longitudinal (viii). Scale bars: A,B,E,F,G,H = 50 μM; C = 25 μM; D = 100 μM. [file 12915_2015_121_MOESM2_ESM.docx]

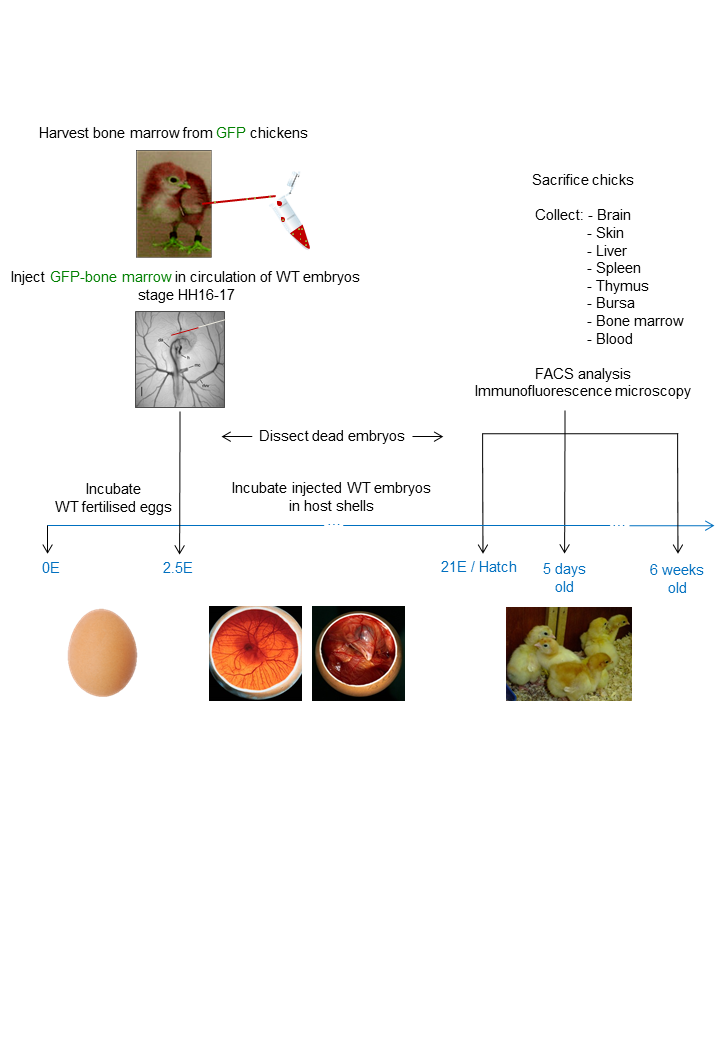


Additional file 2: Figure S1


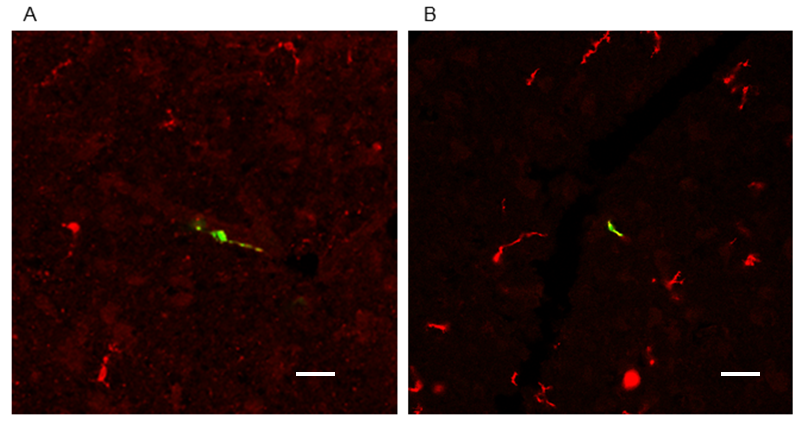


Additional file 2: Figure S2.


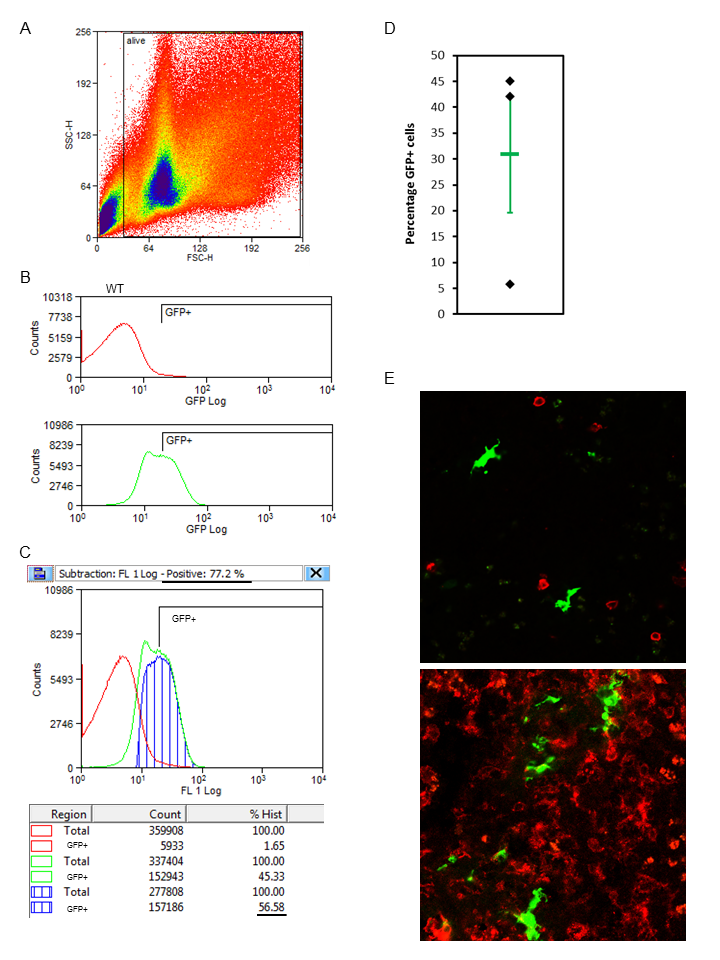


Additional file 2: Figure S**3A**


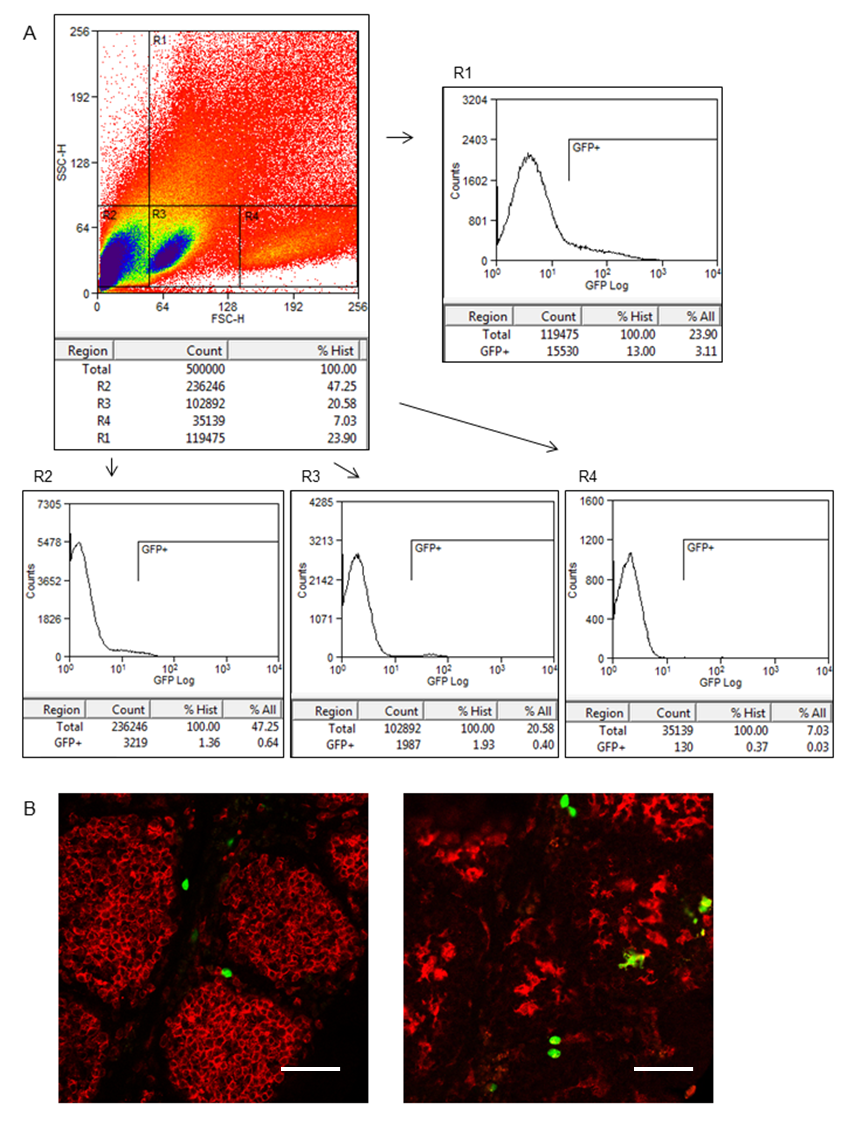
Additional file 2: Figure S**3B.**


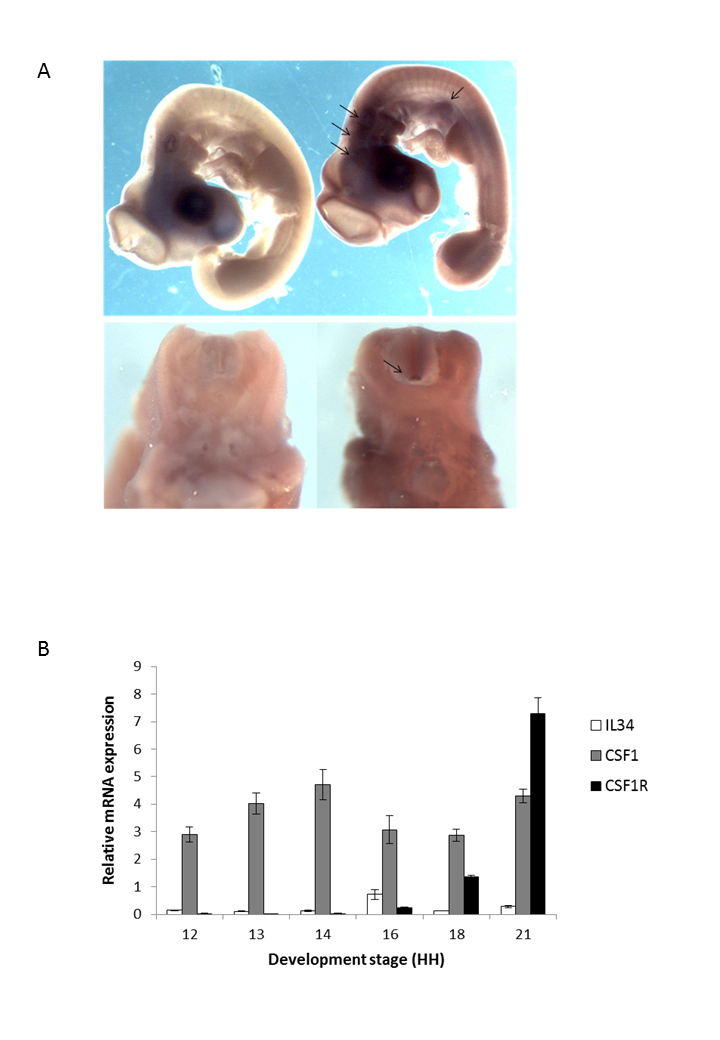


Additional file 2: Figure S**4.**


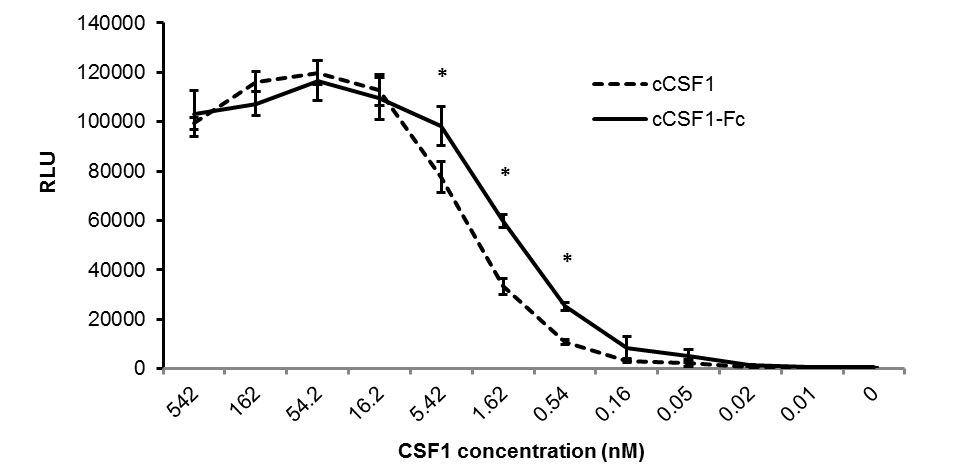


Additional file 2: Figure S**5.**


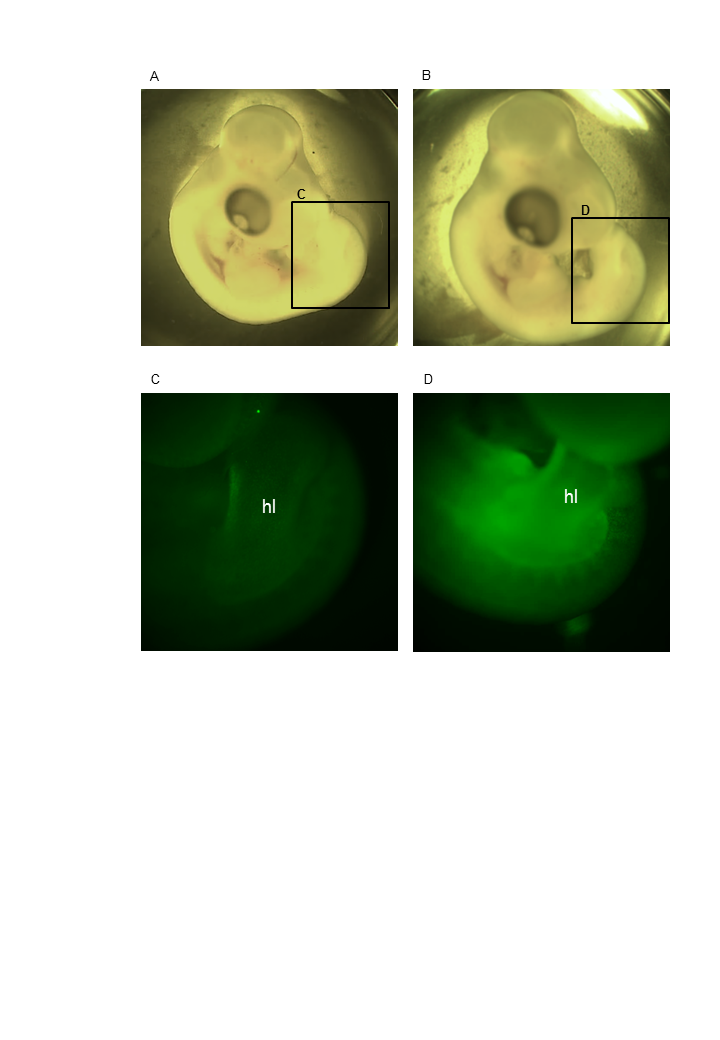


Additional file 2: Figure S6A


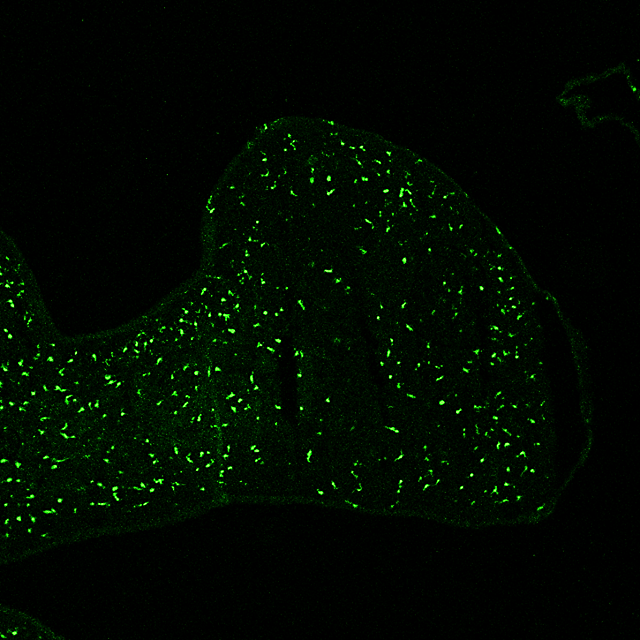

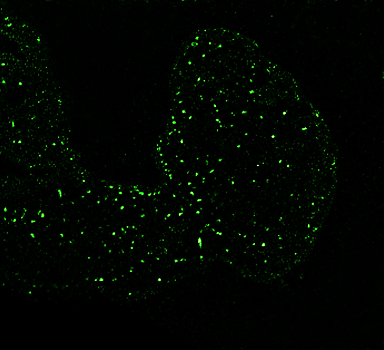

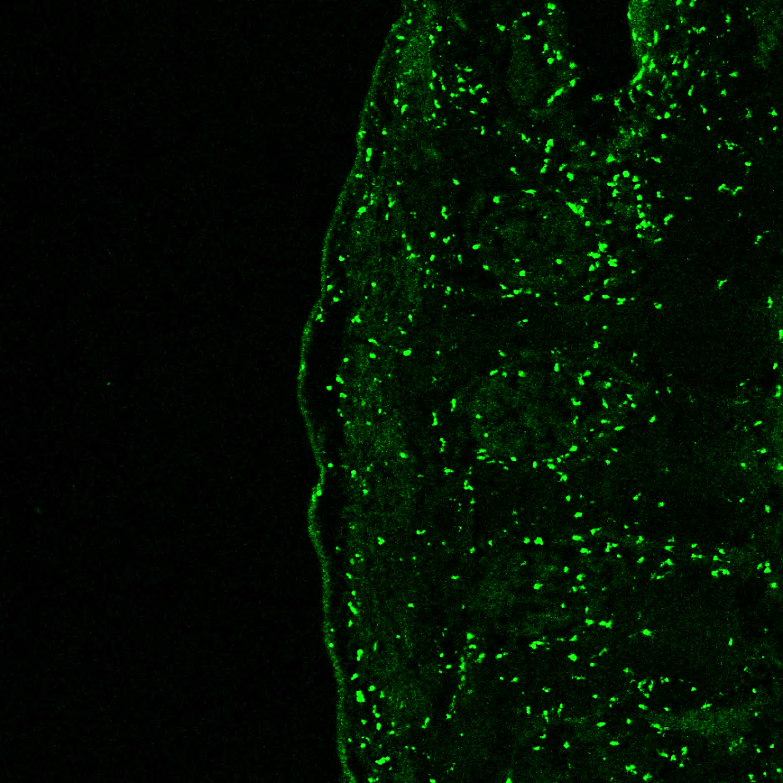

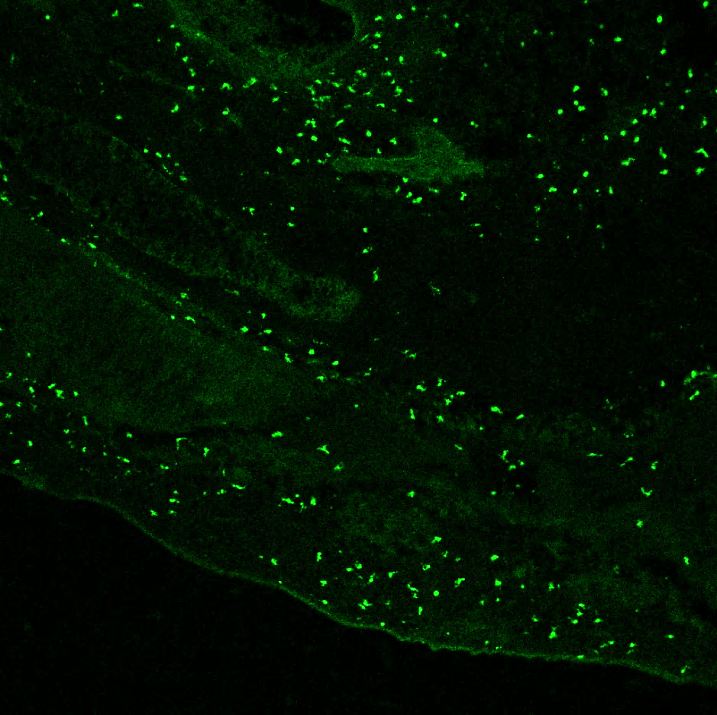


A

B

D

C

S

S

IR

IR

Additional file 2: Figure S6B.


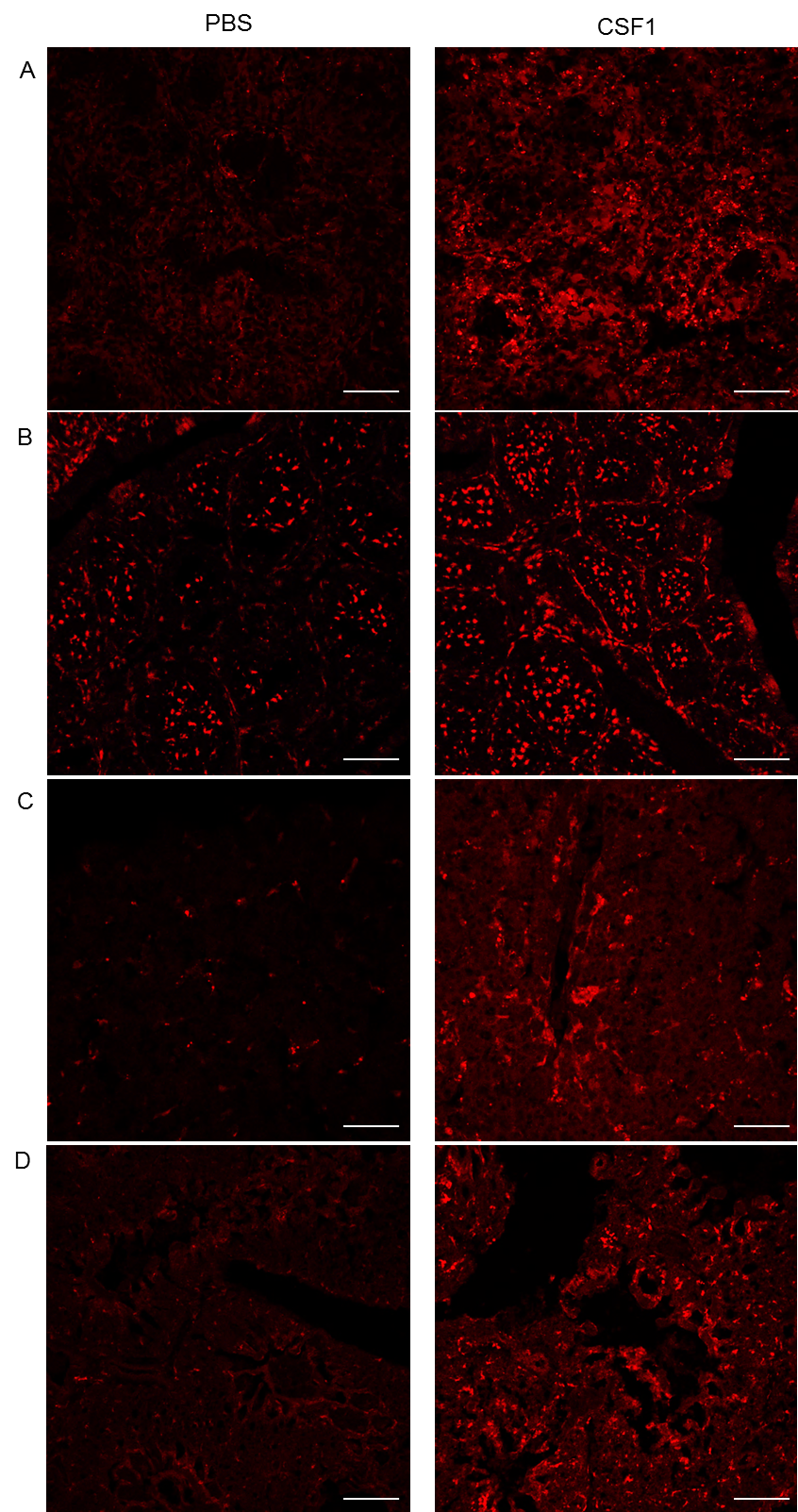


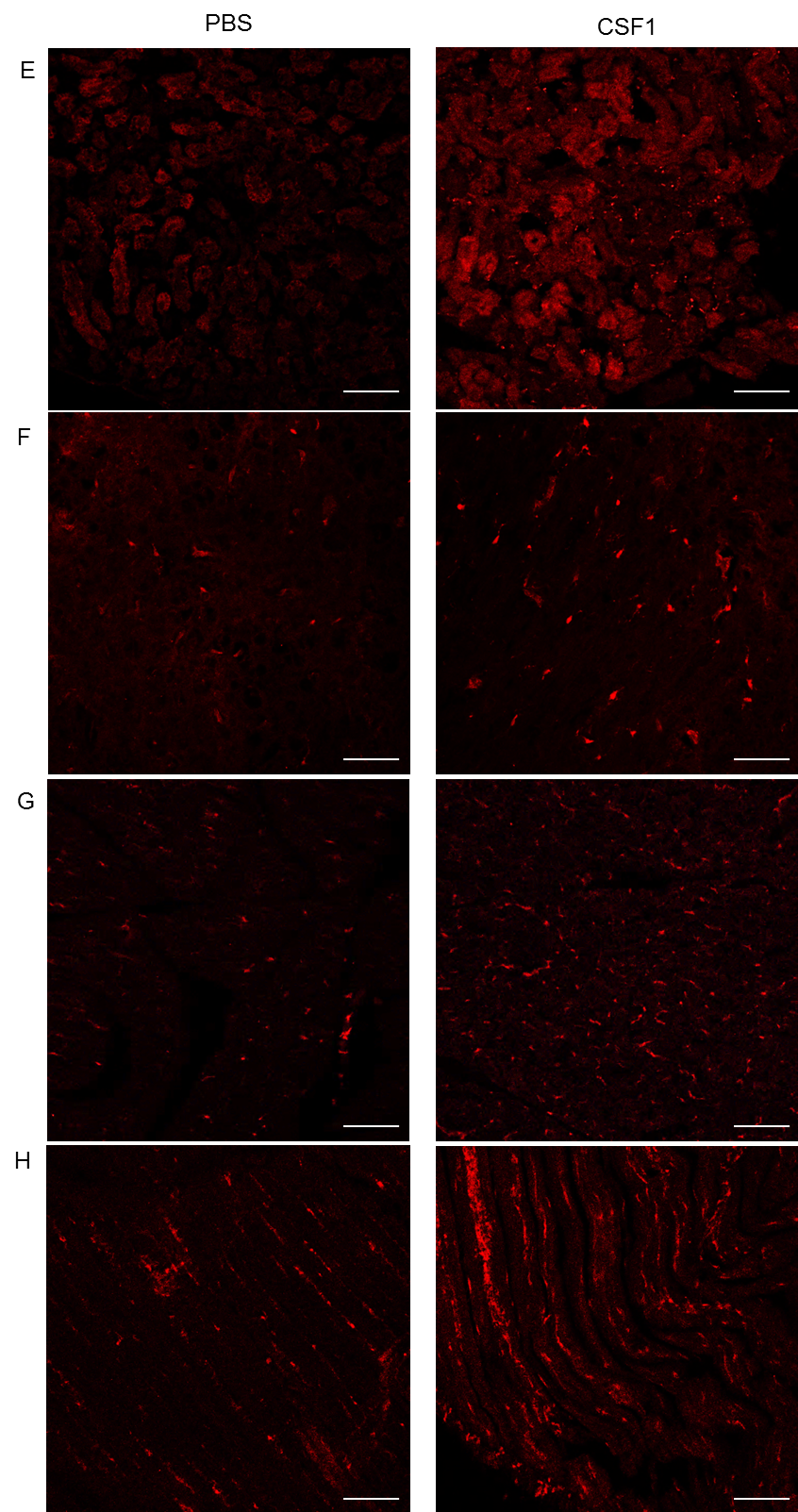
Additional file 2: Figure S7.
